# Unleashing the biological power and chemical profile of *Paracaryum hedgei* extracts based on chromatographic, in vitro, and bioinformatic tools

**DOI:** 10.1016/j.bbrep.2025.102408

**Published:** 2025-12-14

**Authors:** Enver Saka, Sakina Yagi, Bengusu H. Akgul, Eulogio J. Llorent-Martínez, Evren Yildiztugay, Ismail Koyuncu, Meltem Cayci, Ismail Yapıcı, Ilhami Gulcin, Yimao Wu, Meng-Yao Li, Gokhan Zengin

**Affiliations:** a Department of Biology, Science Faculty, Selcuk University, Konya, Turkey; b Department of Botany, Faculty of Science, University of Khartoum, Khartoum, Sudan; c Department of Physical and Analytical Chemistry, University of Jaén, Campus Las Lagunillas S/N, Jaén, 23071, Spain; d Department of Biotechnology, Science Faculty, Selcuk University, Konya, Turkey; e Department of Medical Biochemistry, Faculty of Medicine, Harran University, Sanliurfa, 63290, Turkey; f Harran University, Faculty of Pharmacy, Department of Pharmaceutical Toxicology, Sanlıurfa, Turkey; g Department of Chemistry, Science Faculty, Ataturk University, Erzurum, Turkey; h State Key Laboratory of Systems Medicine for Cancer, Shanghai Cancer Institute, Renji Hospital, Shanghai Jiao Tong University School of Medicine, Shanghai, China; i Shanghai Key Laboratory for Cancer Systems Regulation and Clinical Translation, Shanghai Jiading District Central Hospital, Shanghai, China

**Keywords:** *Paracaryum hedgei*, Radical scavenging, Salvianolic acid, Carbonic anhydrase, Network pharmacology

## Abstract

Studies evaluating the chemical constituents and pharmacological potential of *Paracaryum* species (family Boraginaceae) were limited. The current study was designed to investigate, for the first time, the chemical profile, antioxidant, enzyme-inhibitory, and cytotoxic properties of *P. hedgei* Aytaç & R.R. Mill. Roots extracts recorded the highest total phenolic content (24.72–91.16 mg GAE/g). About 33 compounds were identified, and the aerial parts extracts were dominated by rosmarinic acid, rutin, and sagerinic acid. In general, the root extracts exhibited greater antioxidant activity than the extracts from the aerial parts. Among the solvents, The 70 % EtOH extract of roots showed the highest antiradical (DPPH: 207.90 mg TE/g; ABTS: 238.08 mg TE/g), ion-reducing (FRAP: 384.99 mg TE/g; CUPRAC: 615.27 mg TE/g), and total antioxidant activities (3.21 mmol TE/g), whereas the EtOAc and aqueous extracts from the aerial parts demonstrated the best chelating capacity. The enzyme inhibition varied according to the plant parts examined and the solvents employed for extraction. The EtOAc extract of the roots exerted the highest inhibitory effect towards the human carbonic anhydrase II (IC_50_: 2.32 μg/ml). The cytotoxicity of the extracts was tested against three cancer cell lines (HELA, A549, and HCT-116) and one healthy cell line (HEK-293). Specifically, the EtOAC extracts showed cytotoxic effects on HELA cells sourced from aerial parts (IC_50_: 106.30 μg/ml) and on HCT-116 cells sourced from roots (IC_50_: 96.82 μg/ml). Molecular docking results provided additional support for the enzyme inhibition capabilities of the extracts. Combined network pharmacology and molecular docking analyses revealed that phenolic acids from *P. hedgei* exert anti-cancer effects on cervical and colorectal adenocarcinoma by modulating key pathways, including retinoic acid metabolism, steroid biosynthesis, and inflammatory responses. These findings can provide a scientific starting point for the pharmaceutical potential of *P. hedgei*, and it can be considered a valuable source of natural bioactive compounds for functional nutraceutical and pharmaceutical applications.

## Highlights

• Different extracts of *Paracaryum hedgei* were tested for the first time.
• Thirty-three components were detected in the extracts.
• The hydroalcoholic extract was the most active in the antioxidant assays.
• Ethyl acetate extract exhibited remarkable cytotoxic effect on HELA cell line.
• The plant can be an alternative raw material for therapeutic formulations.

## Graphical abstract

**Figure:**
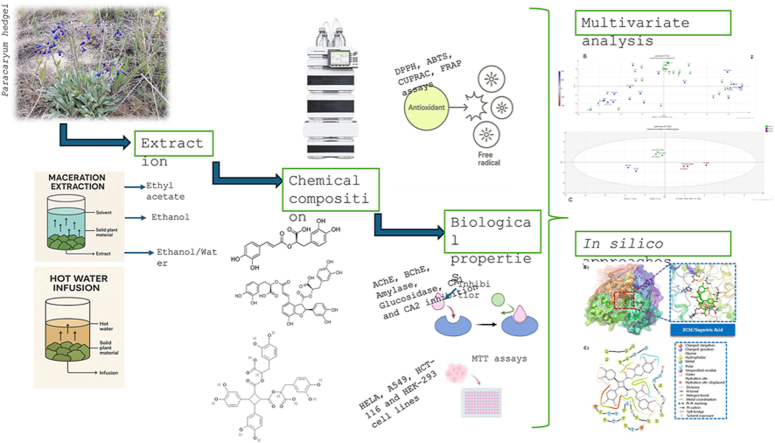

## 1 Introduction

Plants have been used to cure a range of ailments since ancient times. They synthesize diverse metabolites, including phenols, alkaloids, terpenes, and others, which, in turn, exhibit various biological activities, such as antioxidant, antimicrobial, and anticancer properties [[1], [2], [3]]. This wide range of health benefits of plants has provided great inspiration for the discovery of new pharmaceutical leads [4]. Indeed, many plants have yet to be investigated for their pharmacologically active compounds.

The Boraginaceae family is considered a valuable natural resource, comprising genera of great biological importance [5]. Among the genera, the genus *Paracaryum* (DC.) Boiss. (family Boraginaceae) comprised about 67 herbaceous species, mainly distributed in the Irano-Turanian phytogeographical región [6]. In Turkey, about 28 species are recorded, of which 12 are endemic. The genus is divided into three subgenera —Modestomattiastrum, Mattiastrum, and Paracaryum —based on the characteristics of the wing of the nutlet, corolla, and anthers [6]. Few studies evaluating the chemical constituents and pharmacological potential of *Paracaryum* species have been reported. A detailed study of the fatty acid profile of 10 *Paracaryum* species was conducted, and their chemotaxonomic significance was highlighted [7]. Erdogan et al. [8] determined the chemical composition of essential oil from the aerial parts of *P. bingoelianum* and 6,10,14-trimethyl-2-pentadecanone (17.2 %), eucalyptol (12.19 %), *trans*-2-hexanal (8.94 %), germacrene D (6.66 %), and octanal (5.48 %) were identified as the major compounds. The oil exhibited significant antioxidant activity and cytotoxicity against MCF-7 and HT-29 cells [8]. In their screening study of medicinal plants from Jordan and Egypt, Alali et al. [9] and El-Hela et al. [10] reported that *P. rugulosum* had a high total phenolic content, exerted antiradical activity, and, in the latter study, also demonstrated larvicidal activity.

*Paracaryum hedgei* Aytaç & R.R. Mill belongs to the subgen. Mattiastrum is widely distributed in the Inner Anatolian steppes in Turkey. It is a perennial herb with a woody rootstock, producing many stems and sterile rosettes, characterized by distinctive ultramarine blue flowers and smooth nutlets [11].

No prior studies have reported the phytochemical profile or bioactivities of *P. hedgei.* Thus, the current study is the first report and was designed to conduct a comprehensive investigation of the chemical composition, antioxidant, enzyme-inhibitory, and cytotoxic properties of extracts from the aerial parts and roots of *P. hedgei*. The antioxidant activity of the extracts was highlighted by examining their capacity to scavenge free radicals, chelate metal ions, and reduce metal ions. At the same time, their ability to inhibit enzymes was evaluated against acetylcholinesterase, butyrylcholinesterase, tyrosinase, α-amylase, and α-glucosidase, as well as the human carbonic anhydrase isoenzymes I and II. The cytotoxic effects were examined in three cancer cell lines (HELA, A549, and HCT-116) and one healthy cell line (HEK-293). The chemical and biological findings were integrated using *in silico* analysis and network pharmacology methods to gather additional information. The results obtained can fill gaps in the biological and chemical profiles of *P. hedgei*. They can also serve as a scientific starting point.

## 2 Materials and methods

### 2.1 Collection of *Paracaryum hedgei*

In 2023, researchers collected plant specimens in Karapınar, an erosion zone located at an altitude of 1040 m in Konya, Turkey. Dr. Evren Yildiztugay meticulously conducted the taxonomic analysis of the collected samples. A reference specimen, labeled with voucher number EY3391, was officially deposited at the Herbarium of the Science Faculty, Selcuk University, for future verification and study. To preserve the phytochemical properties, the aerial sections and roots were carefully separated into post-collection and dried in a shaded area at ambient temperature. Once dried, the material was finely milled into powder following a standardized procedure. This powdered botanical material was then stored in light-blocking containers under controlled conditions to prevent deterioration and ensure long-lasting preservation.

### 2.2 Extraction protocols

The study compared extracts from four solvents: ethanol, ethyl acetate, a 70 % ethanol-water mixture, and water. The extraction was carried out separately for each solvent. The solvents were analytical grade. The method for the three organic solvents was consistent: 10 g of plant material were macerated in 200 mL of solvent at room temperature over 24 h. The aqueous extract was prepared differently by infusing 10 g of material in hot water (200 ml) for 15 min. Finally, all organic solvents were evaporated under reduced pressure (using a rotary-evaporator at 45 °C), and the water-based extract was lyophilized (freeze-dried, at −80 °C for 72 h). The dried extracts were stored at 4 °C until analysis. Extraction yields are given in Table 1.

**Table 1:** Extraction yields (%), total phenolic and flavonoid content in *Paracaryum hedgeii* aerial parts and roots extracts.

Parts	Solvents	Extraction yields (%)	TPC (mg GAE/g)	TFC (mg RE/g)
Aerial parts	EtOAc	0.70	15.91 ± 0.21^g^	21.44 ± 0.51^c^
	EtOH	6.06	40.45 ± 1.06^e^	40.91 ± 0.96^a^
	70 % EtOH	13.38	57.33 ± 0.95^c^	35.42 ± 0.26^b^
	Water	17.13	53.97 ± 1.13^d^	18.50 ± 0.36^d^

Roots	EtOAc	0.50	24.72 ± 0.28^f^	1.54 ± 0.14^g^
	EtOH	1.64	83.02 ± 0.57^b^	4.67 ± 0.70^e^
	70 % EtOH	4.63	91.16 ± 1.30^a^	3.00 ± 0.11^f^
	Water	4.44	90.90 ± 1.07^a^	5.61 ± 0.04^e^

Values are reported as mean ± SD of three parallel measurements. GAE: Gallic acid equivalent; RE: Rutin equivalent. Different letters in the same column indicate significant differences in the extracts (*p* < 0.05).

**Table 2:** HPLC-ESI-Q-TOF-MS characterization of the compounds found in the analyzed extracts of *Paracaryum hedgeii*.

No.	t_*R*_ (min)	Observed [M − H]^-^	Molecular formula	Error (ppm)	Fragment ions	Assigned identification	Aerial parts	Roots
**1**	1.8	341.1095	C_12_H_22_O_11_	−1.65	**179.0561**, 161.0480, 119.0331	Disaccharide	All	All
**2**	1.9	191.0195	C_6_H_8_O_7_	1.56	**111.0104**	Isocitric acid	EtAc, EtOH:H_2_O, H_2_O	All
**3**	2.0	133.0142	C_4_H_6_O_5_	0.25	**115.0034**	Malic acid	EtOH:H_2_O, H_2_O	All
**4**	2.5	191.0198	C_6_H_8_O_7_	−0.55	**111.0089**	Citric acid	EtAc, EtOH:H_2_O, H_2_O	All
**5**	3.8	197.0453	C_9_H_10_O_5_	1.25	179.0345, **135.0444**, 123.0447	Danshensu	–	All
**6**	3.9	315.0736	C_13_H_16_O_9_	−1.35	**152.0129**, 108.0216	Dihydroxybenzoic acid-*O*-hexoside	EtOH:H_2_O, H_2_O	–
**7**	6.9	337.093	C_16_H_18_O_8_	−0.37	191.0554, 173.0453, **163.0399**, 119.0499	Coumaroylquinic acid	EtOH, EtOH:H_2_O, H_2_O	–
**8**	7.3	137.0241	C_7_H_6_O_3_	1.91	108.0223, 92.0246	Salicylic acid	EtOH, EtOH:H_2_O, H_2_O	All
9	7.5	337.0925	C_16_H_18_O_8_	1,3	191.0555, **163.0396**, 119.0504	Coumaroylquinic acid	EtOH, EtOH:H_2_O, H_2_O	–
10	8.6	153.0193	C_7_H_6_O_4_	0.37	109.0287	Dihydroxybenzoic acid	–	EtAc, EtOH:H_2_O
11	8.9	367.1039	C_17_H_20_O_9_	−1.14	**193,0493**, 134.0342	Feruloylquinic acid	EtOH, EtOH:H_2_O, H_2_O	–
12	11.1	179.0347	C_9_H_8_O_4_	1.67	135.0451	Caffeic acid	All	All
13	12.1	337.0925	C_16_H_18_O_8_	0.78	191.0537, **173.0451**	Coumaroylquinic acid	EtOH, EtOH:H_2_O, H_2_O	–
14	13.7	367.1034	C_17_H_20_O_9_	−0.37	**173.0461**, 93.0331	Feruloylquinic acid	EtOH, EtOH:H_2_O, H_2_O	–
15	17.0	755.2037	C_33_H_40_O_20_	0.38	**300.0266**, 178.9967, 151.0055	Quercetin-*O*-rutinoside-*O*-rhamnoside	EtOH, EtOH:H_2_O	–
16	18.8	458.2027	C_21_H_33_NO_10_	1.03	310.1288, 205.0717, **177.0762**, 131.0709	Unknown	All	EtAc, EtOH, EtOH:H_2_O
17	19.5	537.1037	C_27_H_22_O_12_	−0.29	493.1136, 383.0830, 313.0691, **295.0603**	Salvianolic acid H/I	–	EtOH, EtOH:H_2_O, H_2_O
18	20.5	609.1457	C_27_H_30_O_16_	0.75	301.0342, 178.9982, 151.0033	Rutin	All	–
19	21.5	463.0879	C_21_H_20_O_12_	0.81	**301.0335**, 178.9976, 151.0043	Quercetin-*O*-hexoside	EtOH, EtOH:H_2_O	–
20	22.5	359.0769	C_18_H_16_O_8_	1.01	197.0447, 179.0342, **161.0240**, 133.0296	Rosmarinic acid isomer	EtOH, EtOH:H_2_O	EtOH, EtOH:H_2_O
21	23.6	593.1507	C_27_H_30_O_15_	0.87	**285.0404**, 255.0297, 151.0025	Kaempferol-*O*-rutinoside	All	–
22	24.4	623.161	C_28_H_32_O_16_	1.2	**315.0503**, 271.0233	Isorhamnetin-*O*-rutinoside	EtOH, EtOH:H_2_O	–
23	24.8	447.0929	C_21_H_20_O_11_	0.98	**284.0325**, 255.0282	Kaempferol-*O*-hexoside	EtOH	–
24	25.0	717.1455	C_36_H_30_O_16_	0.8	537.1029, **519.0925**, 493.1133, 339.0508, 321.0400, 295.0606	Salvianolic acid B/E isomer	H_2_O	All
25	25.8	719.1612	C_36_H_32_O_16_	0.66	223.0245, 197.0455, 179.0345, 161.0239	Sagerinic acid	EtOH, EtOH:H_2_O, H_2_O	All
26	25.9	359.0768	C_18_H_16_O_8_	1.03	197.0453, 179.0360, **161.0235**	Rosmarinic acid isomer	All	All
27	27.4	537.1033	C_27_H_22_O_12_	1.0	493.1134, 383.0758, 313.0710, **295.0606**	Salvianolic acid H/I	–	EtOH, EtOH:H_2_O, H_2_O
28	27.9	735.1561	C_36_H_32_O_17_	0.93	555.1150, 357.0605	Unknown	–	EtOH, EtOH:H_2_O, H_2_O
29	29.2	895.1723	C_45_H_36_O_20_	0.38	851.1817, 653.1287, 555.1127, 357.0612, 295.0603	Unknown	–	H_2_O
30	30.1	717.1451	C_36_H_30_O_16_	1.35	**519.0924**, 339.0502, 321.0398, 295.0612	Salvianolic acid B/E isomer	EtOH, EtOH:H_2_O, H_2_O	All
31	34.8	301.0357	C_15_H_10_O_7_	−1.59	178.9990, **151.0034**	Quercetin	EtOH, EtOH:H_2_O	–
32	38.6	327.218	C_18_H_32_O_5_	−1.04	291.1954, 229.1458, **211.1333**, 171.1017	Oxo-dihydroxy-octadecenoic acid	All	EtAc, EtOH, EtOH:H_2_O
33	40.0	329.233	C_18_H_34_O_5_	0.77	311.2245, 229.1446, **211.1345**, 171.1022	Trihydroxy-octadecenoic acid	All	EtAc, EtOH, EtOH:H_2_O

### 2.3 Spectrophotometric methods for total phenolic and flavonoid content

The determination of total phenolic and flavonoid levels in the extracts was performed according to previously established protocols [12]. In the experiments, gallic acid (GA) and rutin (R) served as reference standards, with the outcomes expressed as gallic acid equivalents (GAE) and rutin equivalents (RE). The experimental details are given in the supplemental materials.

### 2.4 Phytochemical analysis by HPLC-ESI-Q-TOF-MS

The compounds were characterized using high-performance liquid chromatography paired with electrospray ionization and a quadrupole time-of-flight mass spectrometer (HPLC-ESI-Q-TOF-MS). For further information on the phytochemical analysis procedures, refer to the supplementary materials.

### 2.5 Antioxidant evaluation

A multi-assay approach was employed to determine antioxidant capacity [13]. The results of the FRAP, CUPRAC, DPPH, and ABTS assays, which evaluate free radical scavenging, are presented as mmol Trolox equivalents (TE) per gram of extract. The phosphomolybdenum (PBD) method for assessing total antioxidant capacity also used mmol TE/g, while the metal chelating activity (MCA) was measured in EDTA equivalents (EDTAE). The experimental details are given in the supplemental materials.

### 2.6 Inhibitory effects on some key enzymes

The samples underwent enzyme inhibition assays utilizing the methods outlined below [13]: AChE and BChE results in galanthamine equivalents (GALAE), amylase and glucosidase inhibition in acarbose equivalents (ACAE), and tyrosinase inhibition in kojic acid equivalents (KAE), all per gram of extract. The analysis of carbonic anhydrase isoenzyme (hCA I and II) inhibition followed a previously published protocol. [14]. The experimental details are given in the supplemental materials.

### 2.7 Cytotoxic effects

#### 2.7.1 Cell culture

Cell lines, both cancerous and normal, obtained from ATCC and stored in liquid nitrogen, were used in the study. These included HELA (Cervical Adenocarcinoma), A549 (Lung Adenocarcinoma), HCT-116 (Colon Adenocarcinoma), and HEK-293 (Human Embryonic Kidney) cells. They were grown in DMEM-F12/RPMI-1640 media supplemented with 10 % Fetal Bovine Serum (FBS), 100 μg/ml streptomycin, and 100 IU/mL penicillin. The cultures were maintained at 37 °C in a humidified incubator with 5 % CO2.

#### 2.7.2 Cell viability assay

We assessed the cytotoxic properties of the extracts using the MTT assay, specifically 3-(4,5-Dimethylthiazol-2-yl)-2,5-Diphenyltetrazolium Bromide. Various cell lines, such as HELA, A549, HCT-116, and HEK-293, were grown in a 96-well sterile plate for a period of 24 h, with each well seeded with 1x10^4^ cells. After removing the growth medium, the extracts were applied at concentrations ranging from 0 to 200 μg/mL for an additional 24 h. Each well then received 10 μL of MTT at a concentration of 0.5 mg/mL as the reactive agent. After a 4-h incubation, the medium was substituted with 100 μL of DMSO, and the absorbance was recorded at wavelengths OD570-OD690 nm using a plate reader (Thermo Multiskan GO, Thermo, USA). Data plots were subsequently generated, and IC50 values were calculated.

#### 2.7.3 Selective index of the extracts in cancer cell compared to the control cell

The study examined the cytotoxic effects of the extracts on several cancer cell lines and determined their IC_50_ values. The findings were contrasted with those observed in both normal healthy cells and cancer cells to compute specific indices. These indices were used to evaluate the extent to which the plant extracts targeted cancer cells. Our analysis assessed the specific indices for eight extracts across three different cancer cell lines: HELA, A549, and HCT-116.

#### 2.7.4 Apoptotic effects by acridine orange/ethidium bromide (AO/EB) staining

At a concentration of 100 μg/ml, AP-EtOAc was administered to HELA cells, and Roots-EtOAc at the same concentration was applied to HCT-116 cells to detect apoptotic morphological changes. After incubation, the cells were washed with PBS and subsequently fixed in 70 % ethanol. After fixation, they were rinsed with distilled water, stained with a working solution of acridine orange/ethidium bromide (Cat No./ID No: A6014-E1510) from Sigma-Aldrich, Germany, and then examined under a fluorescence microscope.

#### 2.7.5 Apoptotic effects by FITC Annexin V assays

The BD Biosciences FITC Annexin V Apoptosis Detection Kit I (New Jersey, USA) was used according to the manufacturer's protocol. HELA and HCT-116 cells were seeded into 6-well plates at a density of 5 × 10^5^ cells per well. After a 24-h incubation, AP-EtOAc and Roots-EtOAc extracts at 100 μg/ml were added, and the cells were incubated for another 24 h. Cells were collected using trypsin and transferred to fresh 1 × 10^6^ tubes containing 1X binding buffer. Each tube was incubated at room temperature for 15 min. Following this, 5 μL each of fluorochrome-tagged Annexin V and Propidium Iodide was introduced. Subsequently, 100 μL of 1X binding buffer was added, followed by centrifugation at 1200 rpm and incubation for another 5 min. The cells were finally analyzed by BD via flow cytometry in New Jersey, USA.

### 2.8 Network pharmacology

#### 2.8.1 Collecting *P. hedgei* potential targets

A comprehensive analysis of the phytochemical constituents of *P. hedgei* was conducted. Utilizing HPLC-ESI-Q-TOF-MS detection results, several compounds were chosen for potential gene target prediction. After identifying key compounds, their chemical structures were sourced from the PubChem database (https://pubchem.ncbi.nlm.nih.gov/) to obtain corresponding CID numbers. These structures were subsequently converted into CID numbers and submitted to both the Swiss Target Prediction database (http://swisstargetprediction.ch/) and the BATMAN-TCM database (http://bionet.ncpsb.org.cn/batman-tcm/#/search) for target prediction analysis. Targets with a prediction probability of zero were eliminated. The predicted targets for each compound were compiled and summarized. The final dataset was visualized using Cytoscape 3.10.2 software. In the network diagram, node color denotes different categories, and node size denotes degree values, with a range of 60–150.

#### 2.8.2 Identification of tumor-associated genes and characterization of their intersection genes

In our pursuit to identify tumor-associated target genes, specifically for cervical adenocarcinoma and colorectal adenocarcinoma, we thoroughly examined several reputable databases: GeneCards (https://www.genecards.org/), Therapeutic Target Database (TTD, https://db.idrblab.net/ttd), DrugBank (https://go.drugbank.com/), and Online Mendelian Inheritance in Man (OMIM, https://omim.org). We used the terms "Cervical Adenocarcinoma" and "Colorectal Adenocarcinoma" as search keywords to fetch pertinent gene targets. The target data sourced from DrugBank was subsequently validated against human-verified proteins listed in the UniProt database (https://www.uniprot.org/). To ensure precision and uniformity, all target data were consolidated, and duplicates were eliminated. The processed compound-gene data and tumor gene expression profiles of cervical adenocarcinoma and colorectal adenocarcinoma have been uploaded to the "Wei-Sheng-Xin" platform (https://www.bioinformatics.com.cn/). Following this, a Venn diagram encompassing compounds, cervical adenocarcinoma, and colorectal adenocarcinoma was constructed to identify overlapping genes among the three categories [15].

#### 2.8.3 Construction of protein-protein interaction (PPI) networks

The STRING database (https://cn.string-db.org/) was used to construct a PPI network to elucidate potential direct and indirect interactions among the identified target proteins. The analysis was restricted to "*Homo sapiens*," and PPI analysis was performed on the intersecting genes of compounds, cervical adenocarcinoma, and colorectal adenocarcinoma. To identify high-confidence interactions, an interaction score threshold of 0.900 was applied, and the PPI network was constructed accordingly. Subsequently, isolated nodes with no connections to other nodes were excluded from the network. The resulting network data were imported into Cytoscape 3.10.2, and the "Analyze Network" function was used to calculate the degree of each node. To visually represent the relative importance of nodes within the network, node size was set to be proportional to each node's degree, with diameters ranging from 60 to 150 pixels. Additionally, node colors were assigned using a continuous gradient color scale based on degree values, thereby enhancing the visual interpretation of node centrality and influence within the network.

### 2.9 Enrichment analysis

The enrichment correlation between *P. hedgei* and two tumor types was analyzed using the Metascape database (http://metascape.org/gp/index.html). The three main categories of GO, namely, biological process (BP), molecular function (MF), and cellular component (CC), were included in the analysis [16]. After obtaining the GO analysis results, the log10-transformed P values were converted back to the original scale, and P ≤ 0.05 was selected. The enrichment scores were ranked in descending order, and the top 10 pathways were selected, categorized, and summarized. Ultimately, the enrichment analysis results were visualized in the Wei-Sheng-Xin platform.

For the Kyoto Encyclopedia of Genes and Genomes (KEGG) analysis, the latest version of KEGG pathway gene annotation data was obtained via the KEGG API (https://www.kegg.jp/kegg/rest/), and this dataset served as the background reference for gene set mapping and subsequent analyses. The functional enrichment analysis of target gene sets was conducted utilizing the "clusterProfiler v3.14.3″ package in R. The minimum and maximum sizes of the gene sets were defined as 5 and 5000, respectively. A P-value threshold of <0.05 and an FDR of <0.25 were adopted as the screening criteria.

### 2.10 Molecular docking

The analysis of *P. hedgei* components aids in identifying compounds that may possess biological activity. These identified compounds are subsequently molecularly docked with selected target proteins to better understand their interaction mechanisms. The selection of target proteins associated with cancer is guided by the results of PPI analysis derived from network pharmacology studies. 3D structural information for these compounds is sourced from the PubChem database (https://pubchem.ncbi.nlm.nih.gov/). The Chem3D v22.2.0 software, paired with the MM2 force field, is utilized to execute energy minimization and optimize molecular conformations. 3D structures of the target proteins are procured from the Protein Data Bank (https://www.rcsb.org/). Protein structures undergo preprocessing using PyMOL v3.1.6.1, a process that includes removing water molecules and native ligands, while incorporating hydrogen atoms. Molecular docking simulations are performed using AutoDock Vina 1.20. The resulting docking data is visualized and scrutinized in Maestro View v14.5, and 2D molecular interaction maps are created to facilitate further interpretation [17].

### 2.11 Molecular dynamics simulation

Molecular dynamics (MD) simulations were conducted using GROMACS 2022.1 with the AMBER14SB force field. The systems were maintained at constant temperature and pressure, with periodic boundary conditions. Each molecule was solvated in a cubic simulation box with dimensions of 10 nm × 10 nm × 10 nm, and the AMBER14SB all-atom force field was consistently applied throughout the simulation. Energy minimization was performed for 50,000 steps using the steepest descent algorithm to remove unfavorable atomic contacts. Subsequently, the NPT ensemble was employed for equilibration and production runs, with the leap-frog algorithm used to integrate the equations of motion. Long-range electrostatic interactions were calculated using the particle mesh Ewald (PME) method. In contrast, van der Waals and Coulombic interactions were truncated at a cutoff distance of 12 Å, with the cutoff updated every 10 steps. Covalent bond lengths involving hydrogen atoms were constrained using the LINCS algorithm with lincs_iter = 1 and lincs_order = 4. Temperature was gradually increased from 0 K to 298.15 K using the V-rescale thermostat, and pressure was maintained at 1 bar using the Parrinello-Rahman barostat to ensure isotropic pressure coupling. Non-bonded interactions were computed using a grid-based neighbor search scheme with a short-range cutoff of 9 Å and a long-range cutoff of 14 Å. Hydrogen bonds were identified based on a donor–acceptor distance less than 0.35 nm and an angle smaller than 30°. Initial velocities were assigned randomly according to the Maxwell-Boltzmann distribution at the target temperature. The total simulation duration amounted to 300 ns, comprising 150,000,000 integration steps with a time step of 2 fs, yielding 10,000 saved conformations for analysis. Trajectories were visualized and analyzed using the built-in tools in GROMACS and Visual Molecular Dynamics (VMD) software [18,19].

### 2.12 Statistical analysis

Each experiment was conducted three times, and statistical comparisons among the extract groups were performed using a one-way ANOVA, followed by Tukey's post hoc multiple-comparison test. Statistical analyses, including ANOVA and Pearson's correlation, were performed using GraphPad Prism (version 9.2). A p-value below 0.05 was considered statistically significant. The relationship between chemical compounds and their bioactivities was evaluated using partial least squares-discriminant analysis (PLS-DA), with SIMCA (version 14.0) used to perform the analysis.

## 3 Results and discussion

### 3.1 Total phenolic (TPC) and flavonoid (TFC) contents

The TPC and TFC of extracts from the aerial parts and roots of *P. hedgei* were determined, and the results are presented in Table 1. The TPC of aerial parts extracts ranged between 15.91 and 57.33 mg GAE/g, and from the roots extracts between 24.72 and 91.16 mg GAE/g. The highest content was recorded in the 70 % EtOH and aqueous extracts of the root (p ≥ 0.05), followed, respectively, by the EtOH extract of the aerial parts and the 70 % EtOH extract of the aerial parts, and the aqueous extract. The EtOAc extract of both organs had the least TPC. The TFC in the aerial parts extract ranged from 18.50 to 40.91 mg RE/g, and in the root extracts, it ranged from 1.54 to 5.61 mg RE/g. Extracts from the aerial parts significantly (*p*˂0.05) accumulated higher TFC than their roots extracts by 13.9-, 8.8-, 11.8-, and 3.3-fold than their respective EtOAc, EtOH, 70 % EtOH, and aqueous roots extracts. Previous studies on the TPC were conducted only on *P. rugulosum*, in which the aqueous and methanolic extracts of a species grown in Jordan were found to contain 22.8 and 15.4 mg GAE/g, respectively [9]. In contrast, those in a species from Egypt were 140.6 % and 98 %, respectively [10].

### 3.2 Phenolic profile by HPLC-ESI-Q-TOF-MS

To identify the compounds, we used analytical standards of caffeic acid, citric acid, isorhamnetin, kaempferol, quercetin, salicylic acid, and rutin. High-resolution mass spectrometry data (accurate mass data and MS/MS fragmentation), bibliographic information, and the METLIN database were also used. More information is provided in the Supplementary Material. Table 2 presents the retention times, experimental [M − H]-, molecular formula, calculated mass error (ppm), and fragment ions for each extract (EtOH, EtOH: H2O, H2O, and ethyl acetate (EtAc)). A summary of the compound characterization is provided; the accurate mass and respective molecular formulas were rigorously verified to ensure precise identification of these compounds. As far as we know, this is the preliminary report on the phytochemical profile of the *Paracaryum* genus. However, the Boraginaceae family, to which it belongs, has been documented to contain danshensu, caffeic acid, rosmarinic acid, salvianolic acids, and glycosides of quercetin and kaempferol as the principal compounds [20], in agreement with our results.

Compound **1** was characterized as a disaccharide (probably diglucoside) due to the fragment ion at *m/z* 341 and its fragmentation pattern [21]; HCl and formate adducts were also observed, so they were taken into account for the relative quantification performed in a future step.

Compounds **2** and **4** were identified as isocitric acid and citric acid, respectively, by comparison with an analytical standard of citric acid (both compounds presented the same fragmentation pattern but different retention times). Compound **3** was characterized as malic acid based on the molecular formula and base peak at *m/z* 115.

Compound **5** was identified as danshensu based on the deprotonated molecular ion at *m/z* 197 and fragment ions at *m/z* 179 and 135 [22].

Compound **6** suffered the neutral loss of 162 Da (hexoside) to yield dihydroxybenzoic acid at *m/z* 152 (base peak at *m/z* 108), so it was tentatively characterized as dihydroxybenzoic acid-*O*-hexoside. Similarly, compound **10**, with a deprotonated molecular ion at *m*/*z* 153, was identified as dihydroxybenzoic acid (a comparison with protocatechuic acid was performed to confirm the molecular fragmentation).

Compounds **7**, **9**, and 13 exhibited [M − H] at *m*/*z* 337, and their molecular formulas and fragmentation patterns were consistent with those of coumaroylquinic acid isomers [23]. Similarly, compounds **11** and **14**, with deprotonated molecular ions at *m/z* 367, were identified as feruloylquinic acid isomers [23].

Compounds **8** and **12** were identified as salicylic acid and caffeic acid, respectively, by comparison with analytical standards.

Several flavonoids (quercetin, kaempferol, and isorhamnetin) were identified in the different extracts. The aglycone quercetin (compound **31**) was determined by comparison with an analytical standard. In contrast, its glycosides (compounds **15**, **18,** and **19**) were characterized by the neutral losses of 146 Da (rhamnoside), 162 Da (hexoside: glucose or galactoside), and 308 Da (rutinoside); in all cases, quercetin was observed at *m/z* 301. Kaempferol glycosides (compounds **21** and **23**) and isorhamnetin-*O*-rutinoside (compound **22**) were characterized by the mentioned neutral losses and the presence of the kaempferol and isorhamnetin aglycones at *m/z* 285 and 315, respectively.

Based on bibliographic information, the fragmentation patterns of compounds **17** and **27**, which had molecular formula C_27_H_22_O_12_, could be attributed to salvianolic acid H or salvianolic acid I [24]. Similarly, compounds **24** and **30**, with molecular formula C_36_H_30_O_16_, could correspond to salvianolic acid B or salvianolic acid E [24,25].

Compound **25** was identified as sagerinic acid based on the molecular formula and fragmentation pattern [26]. Compounds **20** and **26**, with [M − H]^-^ at *m/z* 359 and fragment ions at *m/z* 197, 179, and 161, were characterized as rosmarinic acid isomers [26].

Compounds **32** and **33** were characterized as oxo-dihydroxy-octadecenoic acid and trihydroxy-octadecenoic acid based on bibliographic data [27].

Once phytochemical identification was performed on the different extracts of aerial parts and roots, we quantified the main compounds relative to each other. With this purpose, we constructed heat maps (Table 3, Table 4) using Extracted Ion Chromatograms (EICs) for each compound at its corresponding deprotonated molecular ion; a symmetric expansion of ±5 ppm was used for the EICs. The relative areas of each compound (in percentage, relative to the total area of all compounds in each extract) are shown in Table 3, Table 4

**Table 3:**
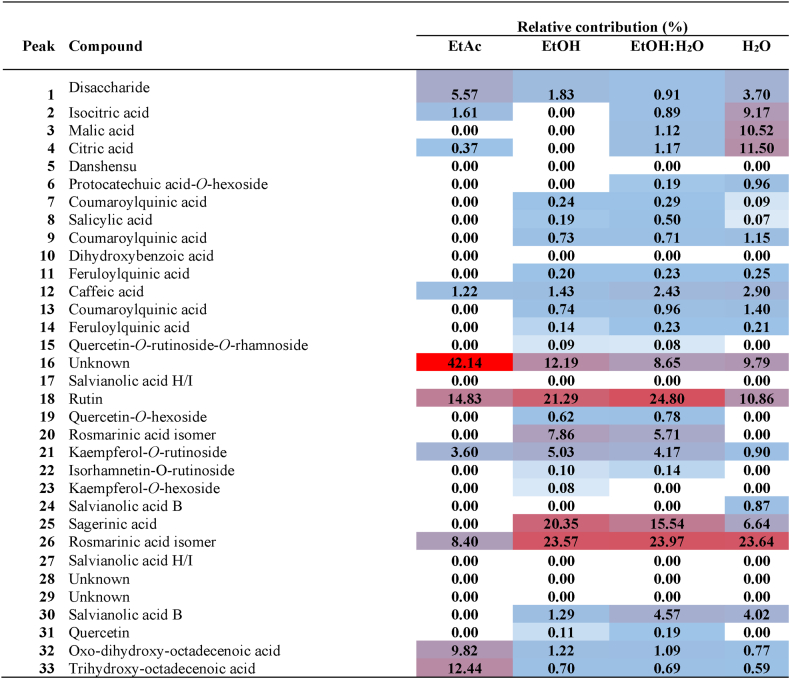
Heat map obtained by HPLC-ESI-Q-TOF-MS of the extracts of aerial parts of *Paracaryum hedgeii*.

**Table 4:**
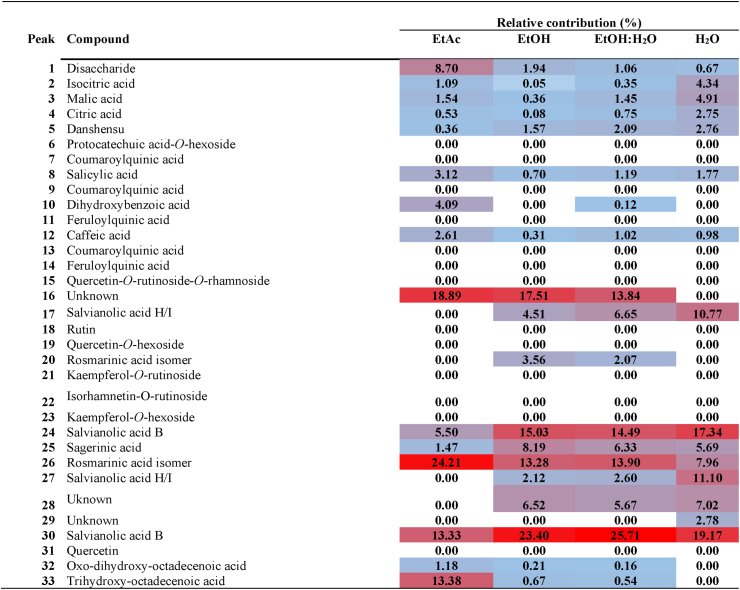
Heat map obtained by HPLC-ESI-Q-TOF-MS of the extracts of roots *Paracaryum hedgeii*.

In the aerial parts, the general profile was similar across all extracts. However, a small number of compounds were detected in the ethyl acetate extract, leading to the identification of an unknown compound (16) with high abundance. In the EtAc extract, the main compounds were the oxylipins **32** and **33**, followed by rutin and rosmarinic acid. In the ethanolic and hydroalcoholic extracts, the main compounds were rutin and rosmarinic acid – each representing more than 20 % of the total extract-, followed by sagerinic acid; these compounds accounted for more than half of the total extract. In the aqueous extract, rutin, rosmarinic acid, and sagerinic acid were also predominant, followed by (iso)citric acids and malic acid. It is worth noting that salvianolic acids, abundant in roots, were absent or present at negligible levels in the aerial parts.

In roots, the main compounds found in the EtOAc extract were rosmarinic acid – more than 20 % -, followed by salvianolic acid B/E and the oxylipin **33**. The composition of the other extracts was similar, observing a high percentage of rosmarinic acid and salvianolic acids B/E (approximately 50 % of the total extract), followed by sagerinic acid. Salvianolic acid H/I was also present in smaller amounts.

### 3.3 Antioxidant activity

Persistent accumulation of reactive oxygen species (ROS) induces cellular oxidative stress, which can lead to pathological damage to cells and, consequently, contribute to the development of chronic diseases. Antioxidants have beneficial health effects as they counteract the generation of ROS through mechanisms such as free radical scavenging, hydrogen donation, metal ion chelation, and others [28]. In the current study, the antioxidant activity of different extracts from *P. hedgei* aerial parts and roots was evaluated using the DPPH, ABTS, CUPRAC, FRAP, MCA, and PBD assays. Results are presented in Table 5. The anti-DPPH and anti-ABTS activities ranged from 0.92 to 207.90 mg TE/g and 8.27 – 238.08 mg TE/g, respectively. The highest effect from both assays was observed in the 70 % EtOH extract from the root, followed by its aqueous extract, and then the 70 % EtOH extract from the aerial parts. The roots also displayed the highest ion-reducing capacity, with values ranging from 64.73 to 615.27 mg TE/g in the CUPRAC assay and from 26.39 to 384.99 mg TE/g in the FRAP assay. In both assays, the highest activity was obtained from the 70 % EtOH and aqueous extracts. The aerial parts extracts were less effective than their corresponding root extracts, and the best effect was observed with the 70 % EtOH extract (CUPRAC = 333.42 mg TE/g and FRAP = 217.70 mg TE/g). Moreover, the root extracts also displayed the highest total antioxidant activity via the phosphomolybdenum assay (1.95–3.21 mmol TE/g), with the best effect recorded from the 70 % EtOH extract, followed by the EtOH and aqueous extracts. Aerial parts extracts displayed lower values (≤1.92 mmol TE/g).

**Table 5:** Antioxidant activity of *Paracaryum hedgeii* aerial parts and roots extracts.

Parts	Extracts	DPPH (mg TE/g)	ABTS (mg TE/g)	CUPRAC (mg TE/g)	FRAP (mg TE/g)	MCA (mg EDTAE/g)	PBD (mmol TE/g)
Aerial parts	EtOAc	0.92 ± 0.05^f^	8.27 ± 1.51^g^	64.73 ± 2.48^g^	26.39 ± 0.68^f^	18.94 ± 0.35^a^	1.74 ± 0.09^d^
	EtOH	45.56 ± 0.21^d^	75.99 ± 1.48^e^	205.70 ± 2.26^e^	120.76 ± 2.69^d^	12.64 ± 0.51^c^	1.75 ± 0.12^cd^
	70 % EtOH	80.74 ± 5.46^c^	133.58 ± 12.59^c^	333.42 ± 2.04^d^	217.70 ± 7.78^c^	14.67 ± 0.47^b^	1.92 ± 0.06^cd^
	Water	43.87 ± 0.10^d^	99.36 ± 0.07^d^	212.12 ± 1.48^e^	129.30 ± 1.01^d^	18.12 ± 0.17^a^	1.52 ± 0.02^e^
Roots	EtOAc	20.56 ± 0.34^e^	40.49 ± 0.98^f^	99.19 ± 4.63^f^	48.75 ± 0.71^e^	1.98 ± 0.19^f^	1.95 ± 0.08^c^
	EtOH	45.77 ± 0.10^d^	99.47 ± 0.13^d^	412.09 ± 4.10^c^	318.08 ± 7.20^b^	5.63 ± 0.08^e^	2.74 ± 0.05^b^
	70 % EtOH	207.90 ± 2.10^a^	238.08 ± 8.71^a^	615.27 ± 9.39^a^	384.99 ± 14.24^a^	10.26 ± 0.32^d^	3.21 ± 0.08^a^
	Water	191.17 ± 6.88^b^	201.13 ± 7.40^b^	564.21 ± 19.33^b^	384.83 ± 9.91^a^	14.29 ± 0.10^b^	2.90 ± 0.05^b^

Values are reported as mean ± SD of three parallel measurements. TE: Trolox equivalent; EDTAE: EDTA equivalent; MCA: Metal chelating activity; PBD: Phosphomolybdenum. Different letters in the same column indicate significant differences in the extracts (*p* < 0.05).

On the other hand, the aerial parts exhibited the highest metal chelating capacity (12.64–18.94 mg EDTA/g), with the highest effect observed in the EtOAc and aqueous extracts (p < 0.05). Many of the identified compounds in the current study were known for their antioxidant property. For example, the rosmarinic acid isomer, accumulated in considerable amounts in extracts of both organs, and rutin, only detected in aerial parts extracts, are well-known compounds for their antioxidant activity [[29], [30], [31]]. Caffeic acid, although it had relatively low abundance, could exert a significant synergistic effect with other antioxidants [32]. However, the relatively high antioxidant activity of the roots could be attributed to the presence of salvianolic acid B and H, which were highly accumulated in the three polar extracts of the roots and were either absent or present at low levels in the aerial parts extracts. In fact, salvianolic acid B was found to exert a significant antiradical effect (EC_50_ = 1.81 and 1.43 μg/mL in the DPPH and ABTS assays respectively) [33].

Furthermore, *in vivo* studies showed that salvianolic acid B effectively alleviated oxidative stress, increased the activities of SOD and GSH-Px, and decreased ROS levels [34]. Salvianolic acid B's highly polyphenolic and conjugated molecular structure gives it remarkable antioxidant properties and enables it to neutralize free radicals in various ways. It has a number of catechol-type hydroxyl groups that readily donate electrons or hydrogen atoms to reactive oxygen species via electron and hydrogen-atom transfer reactions. Resonance-stabilized phenoxyl radicals are created as a result. By facilitating electron delocalization, the extensive π-conjugated system improves redox potential and radical stabilization. Additionally, carboxyl and *ortho*-dihydroxyl groups have a strong metal-chelating ability that effectively sequesters transition metals like Fe^2+^, preventing the formation of Fenton-type radicals [35]. A previous study evaluating the antioxidant activity of *Paracaryum* species included only two species. Essential oil from *P. bingoelianum* exerted anti-DPPH and Fe^+++^ reducing capacity in concentration dependent manner [8]. Aqueous and methanolic extracts of *P. rugulosum* displayed considerable anti-ABTS activity (140.6 and 98.0 μmol TE/g, respectively) [9] and anti-DPPH (52.70 % and 45.30 %) [10]. Thus, it was clear that *Paracaryum* species could be a promising source of antioxidant compounds, and further exploration of the antioxidant activity of other species is recommended.

### 3.4 Enzyme inhibitory activity

Inhibition of enzymes is a key strategy for treating a wide range of diseases. Enzyme inhibitors block the activity of specific enzymes towards their natural physiological substrates without affecting other enzymes. For example, some metabolites inhibit the activity of acetylcholinesterase, an enzyme involved in the pathogenesis of Alzheimer's Disease (AD). Inhibition of tyrosinase, a key enzyme in melatonin biosynthesis, has preventive effects against skin pigmentation disorders [36] as well as Parkinson's Disease. Inhibition of α-glucosidase and α-amylase, key enzymes involved in carbohydrate catabolism, prevents postprandial hyperglycemia [37]. Indeed, there is a continuous need to discover new natural sources of effective enzyme inhibitors. In the present study, the enzyme inhibitory activity of different extracts from the aerial parts and roots of *P. hedgei* was evaluated against acetylcholinesterase (AChE), butyrylcholinesterase (BChE), tyrosinase (Tyr), α-amylase, and α-glucosidase. Results are presented in Table 6. The anti-AChE activity ranged from inactive to 2.99 mg GALAE/g, and that against BChE ranged from 0.73 to 3.54 mg GALAE/g. All extracts, except the aqueous one and the EtOAc extract of aerial parts, possessed comparable effects towards the AChE (*p* ≥ 0.05). However, their anti-BChE activity varied, with the best activity recorded from the EtOH extract of the roots, followed by the EtOH and EtOAc extracts of the aerial parts (*p* ≥ 0.05). The anti-Tyr activity ranged from 18.21 to 63.64 mg KAE/g, with EtOH and 70 % EtOH extracts from both organs showing the highest effect (p ≥ 0.05), followed by their EtOAc extracts (*p* ≥ 0.05). Concerning the enzymes involved in the management of diabetes, it was observed that the extracts were more effective towards ⍺-glucosidase enzyme than ⍺-amylase one, with the EtOH and EtOAc extracts of both organs, in addition to the aqueous extract of the roots, displayed the best inhibitory activity (2.11 – 2.17 mmol ACAE/g, *p* ≥ 0.05). The best inhibitory activity against the ⍺-amylase was recorded from the EtOAc extract of both organs and the EtOH extract of the roots (0.36 – 0.37 mmol ACAE/g). To the best of our knowledge, this is the first report on the enzyme-inhibitory properties of *Paracaryum* species; however, the chemical profile revealed several compounds known for their enzyme-inhibitory activity. For example, rosmarinic acid is an effective inhibitor ofAChE, BChE [38]⍺-glucosidase [39] and tyrosinase [40]. Rutin and caffeic acid inhibit the tyrosinase [41], and the former also inhibits the AChE and ⍺-glucosidase [42]. Kaempferol-3-*O*-rutinoside was reported to be more potent than the reference antidiabetic drug, acarbose towards the ⍺-glucosidase enzyme [43].

**Table 6:** Enzyme inhibitory activity of *Paracaryum hedgeii* aerial parts and roots extracts.

Parts	Extracts	AChE (mg GALAE/g)	BChE (mg GALAE/g)	Tyrosinase (mg KAE/g)	Amylase (mmol ACAE/g)	Glucosidase (mmol ACAE/g)
Aerial parts	EtOAc	na	2.92 ± 0.01^b^	57.05 ± 1.22^b^	0.37 ± 0.01^a^	na
	EtOH	2.90 ± 0.02^a^	2.93 ± 0.12^b^	62.01 ± 0.69^a^	0.32 ± 0.01^b^	2.11 ± 0.01^a^
	70 % EtOH	2.88 ± 0.01^a^	1.82 ± 0.07^d^	63.59 ± 1.16^a^	0.26 ± 0.01^c^	2.14 ± 0.08^a^
	Water	1.28 ± 0.06^b^	0.76 ± 0.13^e^	27.89 ± 2.73^c^	0.10 ± 0.01^d^	1.76 ± 0.10^b^
Roots	EtOAc	2.82 ± 0.20^a^	2.26 ± 0.23^c^	57.78 ± 0.98^b^	0.36 ± 0.01^a^	na
	EtOH	2.97 ± 0.03^a^	3.54 ± 0.08^a^	62.48 ± 0.39^a^	0.36 ± 0.01^a^	2.08 ± 0.03^a^
	70 % EtOH	2.99 ± 0.01^a^	2.57 ± 0.06^c^	63.64 ± 0.69^a^	0.24 ± 0.01^c^	2.17 ± 0.01^a^
	Water	1.40 ± 0.04^b^	0.73 ± 0.03^e^	18.21 ± 2.14^d^	0.07 ± 0.01^e^	2.17 ± 0.02^a^

Values are reported as mean ± SD of three parallel measurements. GALAE: Galantamine equivalent; KAE: Kojic acid equivalent; ACAE: Acarbose equivalent. na: not active. Different letters in the same column indicate significant differences in the extracts (*p* < 0.05).

### 3.5 Inhibitory effects on human carbonic anhydrase isoenzymes I and II (hCA I and hCA II)

Carbonic anhydrases (CAs) are enzymes crucial for regulating pH and metabolism in all living organisms. They play roles in various physiological and pathological processes. Modulating CA activity, either by inhibiting or activating it, has been identified as a promising strategy for diagnosing and treating numerous conditions, including glaucoma, obesity, osteoporosis, ulcers, cancer, Alzheimer's disease, aging, and other neurological disorders [[44], [45], [46]]. The present study evaluated the inhibitory effect of different extracts of *P. hedgei* on the CAI and CAII isoenzymes of human erythrocytes. Results are presented in Table 7. Remarkably, the EtOAc extract of the roots exerted a potent inhibitory effect towards hCAII with an IC_50_ value (2.32 μg/mL) twice higher than that obtained from the standard inhibitor Asetazolamid (IC_50_ 4.81 μg/mL). All other extracts exhibited higher affinity towards CAI (IC50 20.14 – 69.30 μg/mL) compared to CAII (IC50 28.40 – 150.65 μg/mL). Notably, the EtOAc and EtOH extracts were more effective than the other two polar extracts, particularly those derived from the roots.

**Table 7:** Human carbonic anhydrase isoenzymes I and II (hCA I and hCA II) inhibitory property of *Paracaryum hedgeii* aerial parts and roots extracts.

Parts	Extracts	hCA I		hCA II	
		IC_50_ (μg/mL)	R^2^	IC_50_ (μg/mL)	R^2^
Aerial parts	EtOAc	38.07	0.9307	63.00	0.9734
	EtOH	38.08	0.9853	57.75	0.9981
	70 % EtOH	55.44	0.9462	110.00	0.9692
	Water	56.80	0.9431	150.65	0.9384

Roots	EtOAc	20.14	0.9454	2.32	0.9209
	EtOH	23.57	0.9324	28.40	0.9269
	70 % EtOH	28.63	0.9756	55.00	0.9437
	Water	69.30	0.9728	77.86	0.9661
	Asetazolamid (Standart inhibitör)	4.23	0.9836	4.81	0.9940

Studies have shown that, besides sulfonamides/sulfamates/sulfamides, which are known potent CA inhibitors, other compounds, such as salicylic acid and its derivatives, and flavonoids, were also effective CA inhibitors [44]. The inhibitory activity of salicylic acid and its derivatives against the CA isozymes is attributed to the presence of different functional groups (OH and COOH) on their aromatic scaffold [47]. Thus, the potent inhibitory effect towards hCAII exerted by the EtOAc extract of the roots could be partly attributed to the presence of considerable content of salicylic acid.

### 3.6 Cytotoxicity

Cancer is one of the leading causes of death in modern society [48,49]. Although chemotherapy, which uses a variety of chemical agents, is a standard cancer treatment, concerns have been raised about the long-term side effects of these compounds [[50], [51], [52]]. Consequently, there is significant scientific interest in replacing these chemical agents with safer, more effective natural alternatives [[53], [54], [55], [56]]. With this in mind, we investigated the cytotoxic effects of *P. hedgeii* extracts in different cell lines, including HeLa, A549, HCT-116, and HEK-293 (a standard cell line).

Ethyl acetate extracts demonstrated the most potent cytotoxic effects across all tested cell lines. Notably, the root EtOAc extract was highly effective against HCT-116 cells, with an IC_50_ of 96.82 μg/mL, and had lower IC_50_ values for HeLa and A549 cells, approximately 111.29 and 139.32 μg/mL, respectively (Table 8). In contrast, the polar extracts (70 % EtOH and water) consistently showed lower potency, with IC_50_ values ranging from 240 to 410 μg/mL. This suggests that the effects are likely due to the presence of moderate non-polar compounds. A pattern of sensitivity emerged by cell line (HCT-116 > HeLa > A549), and when comparing extracts from different plant parts, root EtOAc extracts were more potent. The overall selectivity indices (SIs) were moderate, with the highest SI of about 1.3 for both root and aerial EtOAc extracts concerning HCT-116 and HeLa cells. Conversely, the hydroalcoholic and aqueous extracts mostly showed SIs ≤1, indicating limited ability to differentiate between cancerous and HEK-293 cells. In summary, these findings highlight the EtOAc fractions, especially those derived from roots, as key candidates for further bioassay-guided fractionation to isolate active components and potentially enhance selectivity.

**Table 8:** Cytotoxic effect (IC_50_ (μg/ml)) of the tested extracts and cisplatin and selective index (SI) evaluation of cancerous cells compared to HEK-293 normal cells.

Parts	Extracts	HELA		A549		HCT-116		HEK-293
		IC50	SI	IC50	SI	IC50	SI	IC50
Aerial parts	EtOAc	106.307	1.3	141.56	1.0	118.09	1.2	142.32
	EtOH	345.65	0.8	366.98	0.8	310.23	0.9	290.59
	70 % EtOH	247.37	1.0	256.89	0.9	282.11	0.9	243.03
	Water	298.21	0.8	321.32	0.8	289.72	0.8	245.88

Roots	EtOAc	111.288	1.1	139.32	0.9	96.823	1.3	125.044
	EtOH	178.09	0.9	201.87	0.8	165.32	0.9	154.87
	70 % EtOH	401.23	0.7	387.49	0.7	367.099	0.8	290.31
	Water	390.98	0.8	410.41	0.8	365.82	0.9	312.49
Cisplatin		12.54	0.3	6.541	0.6	5.124	0.8	4.24

**Fig. 1:**
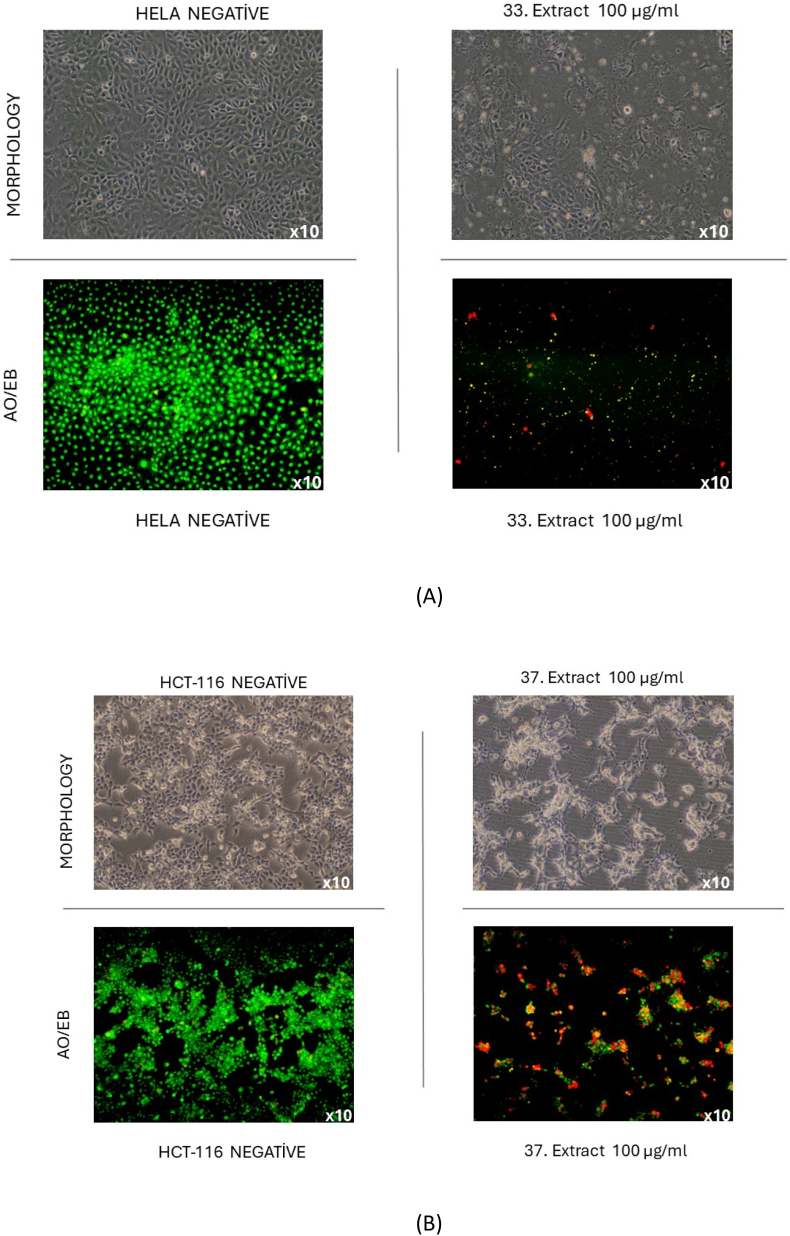
(A) AO/EB staining after Aerial parts-EA (100 μg/ml) applied to HELA cell. (B)AO/EB staining after Roots-EA (100 μg/ml) applied to HCT-116 cell.

Collectively, the morphological evaluation and AO/EB staining indicate the pronounced cytotoxic effects of both tested compounds on HeLa and HCT-116 cells at 100 μg/mL (Fig. 1). In the untreated control group, the cells retained their typical morphology, displaying high confluency and intact membrane integrity as evidenced by uniform green fluorescence (viable cells). In contrast, treatment with aerial parts - EtOAc extract markedly disrupted the morphology of HeLa cells, reducing cell density and inducing shrinkage, rounding, and detachment. AO/EB staining confirmed extensive cell death, with increased red/orange fluorescence indicating late apoptosis and necrosis. Similarly, exposure of HCT-116 cells to the roots EtOAc extract caused severe morphological alterations, including cell rounding and detachment, along with a pronounced increase in red/orange fluorescence. The fact suggests compromised membrane integrity and an apoptotic/necrotic transition. These findings demonstrate that both compounds exhibit strong cytotoxicity through membrane damage and apoptotic pathways [57]. Visible phenotypic and fluorescent evidence support their potential as cytotoxic agents.

**Fig. 2:**
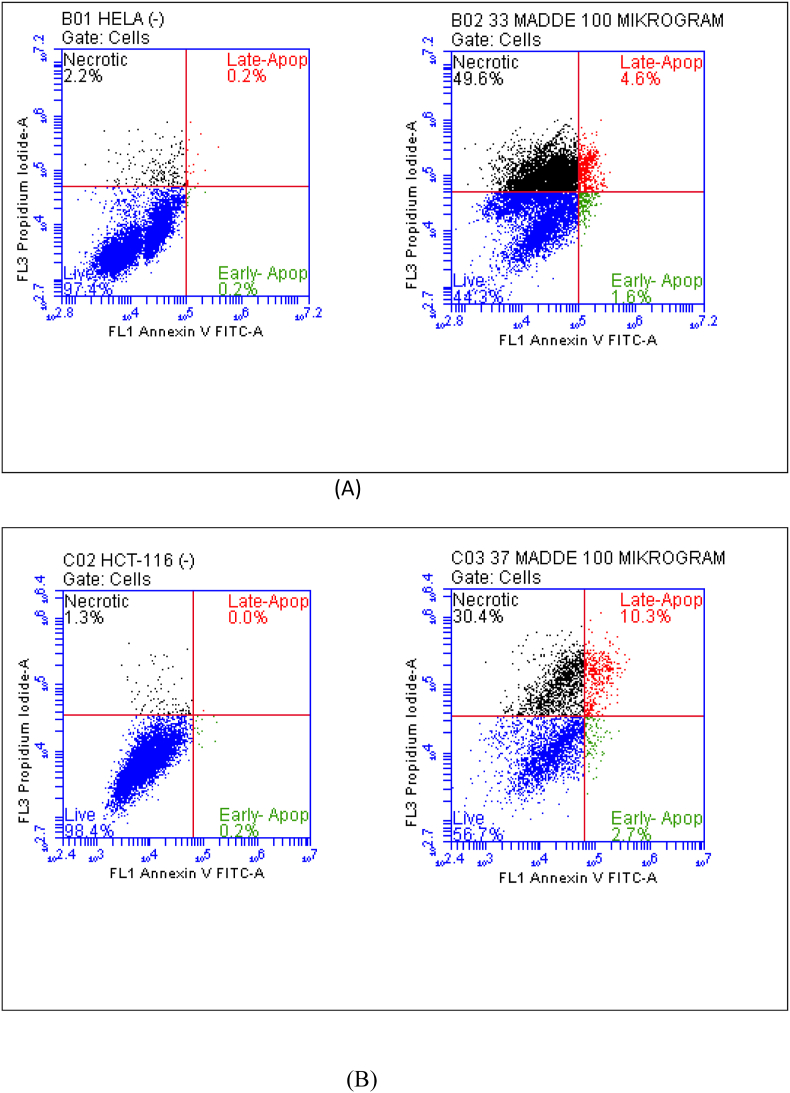
(A) Annexin-V/PI staining results after Aerial parts-EA (100 μg/ml to HELA cell. Blue: Live-[(FITC-)/(PI-)]; Green: Early apoptotic [(FITC+)/(PI-)]; Red: Late apoptotic [(FITC+)/(PI+)]; Black: Shows necrotic [(FITC+)/(PI+)] cells. (B) Annexin-V/PI staining results after Roots-EA (100 μg/ml to HCT-116 cell. Blue: Live-[(FITC-)/(PI-)]; Green: Early apoptotic [(FITC+)/(PI-)]; Red: Late apoptotic [(FITC+)/(PI+)]; Black: Shows necrotic [(FITC+)/(PI+)] cells.

Annexin V and propidium iodide (PI)-based flow cytometric analysis showed a significant increase in the percentage of viable cells after administering both test compounds at 100 μg/mL (Fig. 2). In the untreated HeLa and HCT-116 control groups, over 97 % of cells remained viable, with necrotic or apoptotic cells making up less than 2 %. Treatment with aerial parts —EtOAc extract — decreased the proportion of viable HeLa cells to 44.6 %, increased the proportion of necrotic cells to 49.6 %, and led to a mild increase in late apoptosis to 4.6 %, pointing to primarily necrotic cell death. Similarly, exposure to roots- EtOAc extract reduced the percentage of live HCT-116 cells to 56.7 %, with necrosis increasing to 30.4 % and late apoptosis to 10.3 %, indicating a combination of necrotic and apoptotic cytotoxic effects. These findings support the morphological observations and AO/EB staining results, confirming that both compounds exhibit significant cytotoxicity by disrupting cell membranes and triggering cell death programs, with variations in the mode of cell death depending on the cell line.

In the literature, information on the cytotoxic effects of members of the genus *Paracaryum* is very scarce. For example, in a previous study by Erdogan et al. [8], the cytotoxicity of P. bingoelianum essential oil was tested on MCF-7 and HT-29 cell lines, and it induced apoptosis in these cell lines. Based on the chemical profiles of the tested extracts, the observed cytotoxic effects can be attributed to specific components. For example, rutin is known to be a potent anticancer and apoptotic agent [58]. In addition, rosmarinic acid exhibits a remarkable cytotoxic effect on several cell lines and has been shown to induce apoptotic pathways [59]. Salvianolic acid B has also been reported as an anticancer agent, inhibiting cell proliferation, inducing apoptosis, and arresting the cell cycle. In this sense, *P. hedge*ii can be considered a natural and effective cytotoxic agent in the preparation of medicine, rather than a chemoprotective agent [60].

### 3.7 Multivariate analysis

**Fig. 3:**
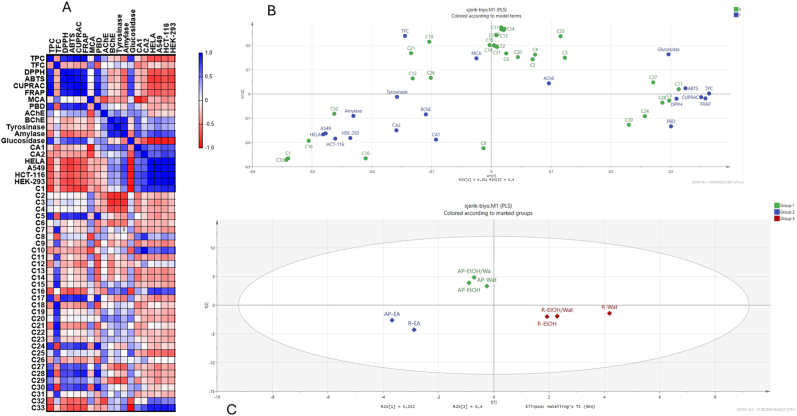
(A) Pearson correlation between chemical compounds and biological activities. (B) Scatter plot for chemical compounds and biological activities from PLS-DA analysis. (C) Biplot distribution based on chemical compounds (based on Table 2) and biological activities of the tested extracts.

Through multivariate analysis, we gain a deeper understanding of the interactions between chemical components and the biological activities of plant extracts. Consequently, we carried out Pearson correlation and PLS-DA analysis, the results of which are depicted in Fig. 3. The Pearson correlation heat map revealed a strong association between radical scavenging (DPPH and ABTS) and reducing power (CUPRAC, FRAP, and phosphomolybdenum) with total phenolic content. This suggests that phenolic compounds play a key role as electron and hydrogen donors in the tested extracts. Conversely, enzyme inhibition assays indicated that, apart from glucosidase, there was no correlation with total phenolic content. Notably, glucosidase inhibition was linked to C25 (sagerinic acid). This fact can be linked to its abundant polyphenolic structure of sagerinic acid, which includes numerous hydroxyl and carboxyl groups. These groups can form strong hydrogen bonds with the enzyme's catalytic residues. The presence of conjugated caffeic acid units promotes π–π stacking and electrostatic interactions, thereby obstructing substrate binding and catalytic activity. In addition, CA1 and CA2 inhibition were linked to C8 (salicylic acid) and C10 (dihydroxybenzoic acid), respectively. Some researchers have identified these compounds as potent carbonic anhydrase inhibitors [61,62]. The cytotoxic effects observed in the tested cell lines were associated with C10, C16, C32, and C33. The Pearson correlation findings were corroborated by PLS-DA analysis, as illustrated in the scatter plot in Fig. 3B. Based on their chemical constituents and biological properties, the extracts were categorized into three groups. Polar extracts from both parts were grouped separately, while ethyl acetate extracts from both parts fell into the same category (Fig. 3C). Overall, the choice of solvent and plant parts emerges as a critical factor in designing functional applications using *P. hedgeii*, providing a scientific foundation for future research.

### 3.8 Network pharmacology

**Fig. 4:**
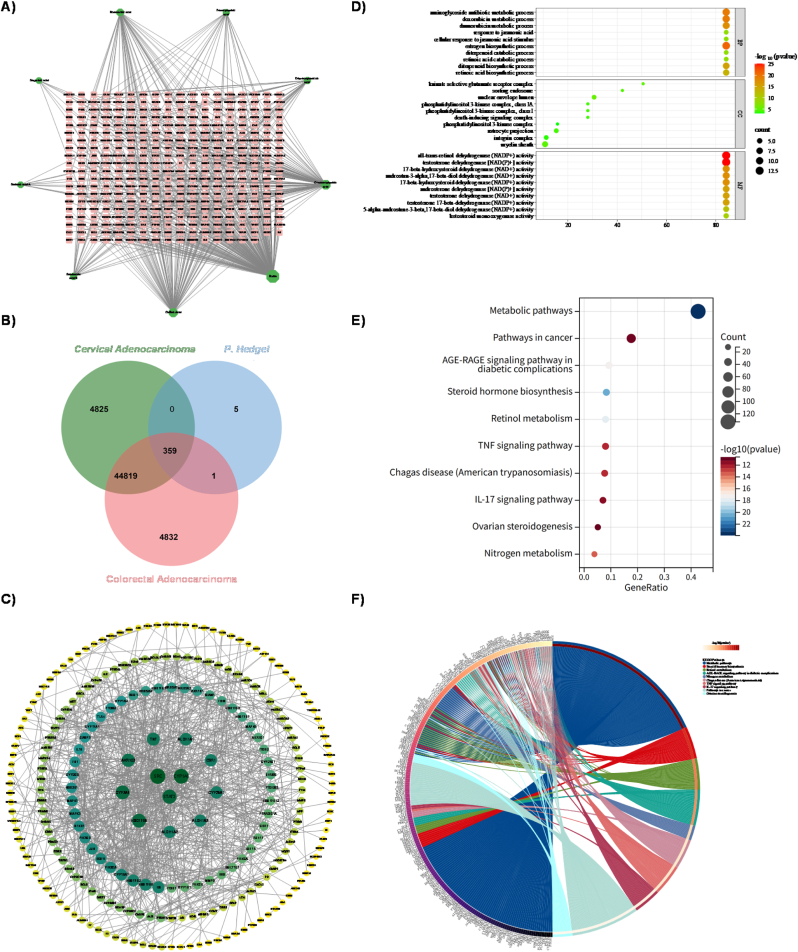
Network pharmacology and functional enrichment analysis of *P*. *hedgei* compounds in cervical adenocarcinoma and colorectal adenocarcinoma. **A)** The active compounds-targets network. **B)** The Venn diagram shows 359 common targets of *P. hedgei* compounds in the two cancer. **C)** The PPI network of 359 co-targeted genes/proteins constructed using STRING. **D)** The GO enrichment analysis results for the targets of the two cancer. **E)** The Top 10 KEGG pathways enriched in the two cancer. **F)** The KEGG chord diagram.

The component analysis of *P. hedgei* extract revealed nine phenolic acid compounds that were subsequently selected for more detailed investigation: caffeic acid, coumaroylquinic acid, dihydroxybenzoic acid, feruloylquinic acid, rosmarinic acid, rutin, sagerinic acid, salianic acid A, and salvianolic acid B. This analysis identified 365 potential targets associated with *P. hedgei* (Fig. 4A). Based on results from cell experiments, cervical adenocarcinoma [63] and colorectal adenocarcinoma were selected for a focused network pharmacology analysis [64,65]. For these two cancer types, related genes were sourced from the GeneCards, TTD, DrugBank, and OMIM databases. After removing duplicate entries, a final list of 50,003 genes associated with cervical adenocarcinoma and 50,011 genes linked to colorectal adenocarcinoma was compiled. The targets derived from *P. hedgei* were then cross-referenced with the gene sets, yielding 359 common targets (Fig. 4B).

We conducted a PPI analysis based on the 359 identified target proteins. The network consisted of 244 nodes and 854 edges (Fig. 4C). Node coloration was based on degree centrality values, ranging from green (higher values) to yellow (lower values), thereby depicting the relative significance of each node within the network. Further analysis identified several hub genes —SRC, STAT3, and CYP1A1 —that may function as central regulators in disease-associated pathways and possess significant biological relevance.

The GO enrichment analysis of the common targets of the *P. hedgei* extract in both cervical and colorectal adenocarcinoma demonstrated significant enrichment of pathways and functional categories (p ≤ 0.05; Fig. 4D). The associated biological processes were chiefly related to retinoic acid metabolism and biosynthesis, specifically the 9-cis-retinoic acid biosynthetic pathway. Additionally, there was an indication of the regulation of vitamin D biosynthesis. These findings suggest that *P. hedgei* may impact cell differentiation and proliferation via retinoid and vitamin signaling pathways. Analysis of the cellular component revealed key signaling complexes, including the death-inducing signaling complex and the phosphatidylinositol 3-kinase complex, suggesting roles in apoptotic regulation and modulation of the PI3K pathway. The molecular function analysis primarily focused on steroid hormone metabolism, with notable activities including 17-beta-hydroxysteroid dehydrogenase (NAD+) activity, testosterone dehydrogenase (NAD+) activity, all-trans-retinol dehydrogenase (NADP+) activity, and all-trans-retinol binding. This comprehensive functional profile strongly indicates that the anti-adenocarcinoma effects of *P. hedgei* extract are likely mediated through its multi-target influence on critical metabolic pathways [66]—particularly those involved in retinoic acid synthesis and steroid hormone transformation—which play essential roles in regulating cancer cell growth and survival.

Of the 359 common targets analyzed, KEGG pathway enrichment identified 165 significant pathways (p ≤ 0.05), with the top 10 pathways illustrating the diverse anti-adenocarcinoma mechanisms of *P. hedgei* extract. The enrichment of Metabolic pathways, Steroid hormone biosynthesis, and Retinol metabolism corroborates the previous GO analysis, highlighting a central role in metabolic reprogramming—particularly in retinoid and steroid hormone signaling—both of which are critical for regulating cell differentiation and proliferation. Additionally, the significant enrichment of the AGE-RAGE signaling pathway in diabetic complications, TNF signaling pathway, and IL-17 signaling pathway suggests a strong immunomodulatory and anti-inflammatory effect [67,68], potentially inhibiting the tumor-promoting microenvironment [69,70]. The overlap between these metabolic and immunoinflammatory pathways and broad oncogenic processes, as demonstrated by the enrichment of Pathways in cancer and specific mechanisms such as ovarian steroidogenesis, indicates that *P. hedgei* extract exerts its anti-cancer effects through a coordinated dual targeting of tumor cell metabolism and host inflammatory responses, offering a multi-targeted therapeutic approach against both cervical [71] and colorectal [72] adenocarcinoma (Fig. 4E and F) [73].

### 3.9 Molecular docking

**Fig. 5:**
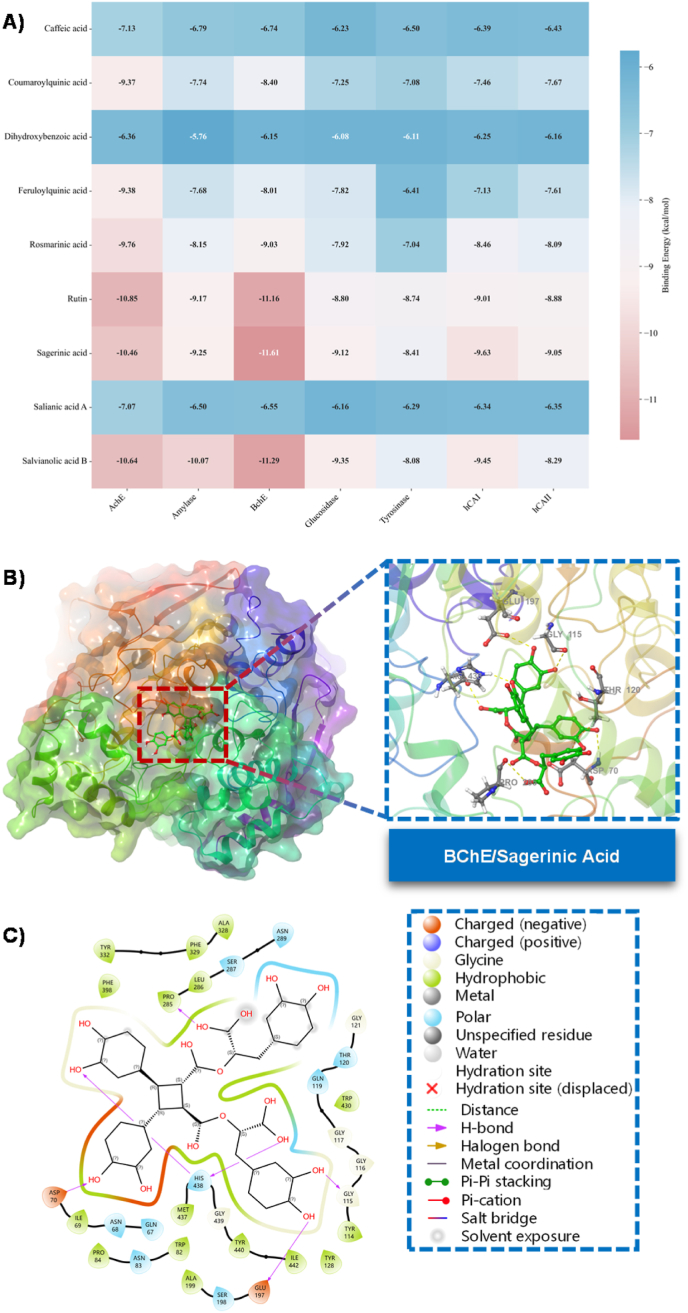
The docking results and correlation analysis of nine compounds from *P. hedgei* against seven metabolic enzymes. **A)** Docking ΔG heat-map (kcal/mol). **B)** Binding mode of sagerinic acid in the BChE active site. **C)** The 2D representation of the binding pockets of BChE with sagerinic acid.

We utilized molecular docking techniques to comprehensively assess the interactions between nine phenolic compounds (caffeic acid, coumaroylquinic acid, dihydroxybenzoic acid, feruloylquinic acid, rosmarinic acid, rutin, sagerinic acid, salianic acid A, salvianolic acid B) and seven pivotal drug targets (AchE, BchE, Amylase, Glucosidase, hCAI, hCAII, Tyrosinase). The binding energy (kcal/mol) is a crucial metric for determining binding strength and potential inhibitory efficacy (Fig. 5). Our findings reveal that rutin, sagerinic acid, and salvianolic acid B demonstrate the most potent binding capabilities across all examined enzyme systems. Notably, their binding energies were consistently below −8.5 kcal/mol, and even surpassed −10 kcal/mol for several targets. This may suggest that these three compounds possess significant multi-target inhibitory potential. Conversely, simpler monophenolic compounds such as caffeic acid, dihydroxybenzoic acid, and salianic acid A exhibited generally higher binding energies with each target, indicative of comparatively weaker binding affinities.

AChE and BChE serve as crucial therapeutic targets for AD. The data revealed that the binding energies of all compounds with BChE were consistently lower than those with AChE (for instance, sagerinic acid: −11.61 vs. −10.46; salvianolic acid B: −11.29 vs. −10.64). This suggests that these compounds may have a higher affinity and selectivity for BChE. This trend aligns with the existing literature, which indicates that the active cavity of BChE is generally larger than that of AChE, enabling it to accommodate high-molecular-weight polyphenolic compounds more effectively. Rutin, sagerinic acid, and salvianolic acid B demonstrated powerful binding capabilities (binding energy < −10 kcal/mol) to both ChEs, implying potential significant anti-cholinesterase activity.

Amylase and glucosidase are integral therapeutic targets for diabetes due to their capacity to regulate postprandial blood glucose levels. The binding energy of salvianolic acid B to amylase was found to be as low as −10.07 kcal/mol, marking it as the most potent among all tested compounds and significantly outperforming the positive control drug acarbose, which typically exhibits binding energies between −8.0 and −9.0 kcal/mol. Similarly, salvianolic acid B demonstrated an optimal effect on the binding energy of glucosidase (−9.354 kcal/mol). This robust enzyme-binding capability can be attributed to its large molecular structure and the presence of multiple phenolic hydroxyl groups, which can form hydrogen bonds and π-π stacking interactions with amino acid residues in enzyme active sites.

The binding affinities of compounds to hCA I and hCA II exhibit notable similarities, albeit with minor differences in binding strength. Notably, sagerinic acid and salvianolic acid B demonstrated the most favorable binding energy to hCA I. Conversely, rutin exhibited a pronounced binding affinity to hCA II, registering at −8.885 kcal/mol. Tyrosinase, pivotal for melanin synthesis in the skin, is the primary target for whitening agents and anti-hyperpigmentation therapeutics. When considering this target, the majority of the compounds showed lower binding energy than for other targets, with Rutin recording the most significant value at −8.742 kcal/mol.

Our molecular docking study suggests that several polyphenolic acids derived from natural sources, notably rutin, sagerinic acid, and salvianolic acid B, exhibit pronounced binding affinities to a range of disease-associated enzyme targets. These compounds present significant potential as multi-target inhibitors for conditions such as AD, diabetes and its complications, glaucoma, among others. Their robust binding is attributed to their extensive molecular structures, rich phenolic hydroxyl groups, and adaptable conformations. Future research should prioritize the experimental validation of these potent compounds and delve deeper into their specific inhibitory mechanisms and *in vivo* effectiveness.

**Fig. 6:**
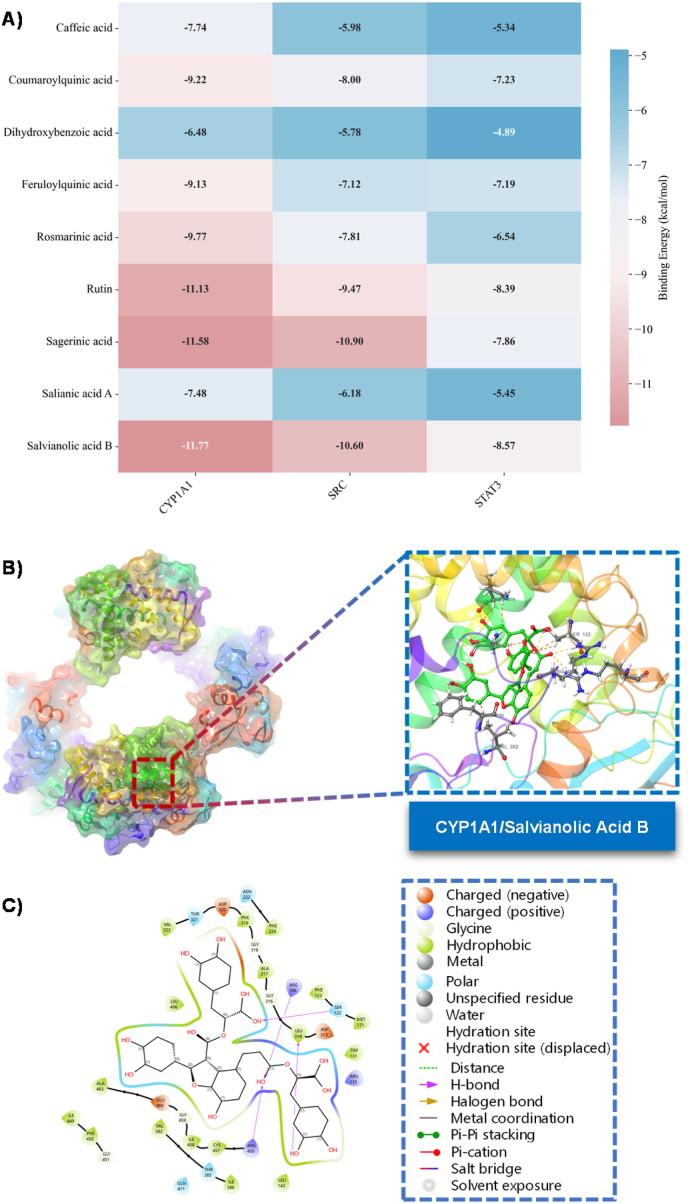
The docking results and correlation analysis of nine compounds from *P. hedgei* against seven metabolic enzymes. **A)** Docking ΔG heat-map (kcal/mol). **B)** Binding mode of salvianolic acid B in the CYP1A1 active site. **C)** The 2D representation of the binding pockets of CYP1A1 with salvianolic acid B.

Moreover, based on the network pharmacology results, we used molecular docking to assess the binding affinities of various phenolic acids for key tumor target proteins (CYP1A1, SRC, and STAT3). The findings revealed that all examined compounds exhibited commendable binding activity on the three target proteins, with binding energies below −5.0 kcal/mol. This suggests that the binding process may occur spontaneously. Notably, salvianolic acid B, sagerinic acid, and rutin demonstrated exceptional performance, registering the lowest binding energies of −11.77, −11.58, and −11.13 kcal/mol against CYP1A1, respectively. These values indicate a significantly high affinity for the target protein. Furthermore, against the non-receptor tyrosine kinase targets SRC and STAT3, both salvianolic acid B and sagerinic acid exhibited the most potent binding potential, with their binding energies surpassing −10.0 kcal/mol—a marked improvement over other small molecule compounds (Fig. 6).

These findings have significant pharmacological potential. CYP1A1, a key enzyme involved in drug metabolism, can inhibit the bioactivation of chemicals. Similarly, SRC and STAT3, central regulatory nodes in cancer signaling pathways, are overactivated and are closely linked to tumor proliferation, survival, and metastasis [74]. The phenolic compounds under discussion demonstrate efficient binding with these targets, suggesting they may directly inhibit the activity of these crucial proteins, thereby regulating the transmission of carcinogenic signals. Consistent with network pharmacology analyses, molecular docking results indicate that natural compounds in *P. hedgei* may directly interact with key signaling proteins. These compounds could synergistically regulate metabolic reprogramming and inflammatory responses in the tumor microenvironment, effectively inhibiting the progression of adenocarcinoma [75]. It is important to note that these molecular docking results provide a theoretical model for the potential interactions between the dominant phytochemicals of *P. hedgei* and the target proteins. The strong binding affinities predicted for compounds such as rutin, sagerinic acid, and salvianolic acid B provide a plausible structural basis for the significant enzyme-inhibitory and cytotoxic activities observed in vitro and warrant further experimental validation.

### 3.10 Molecular dynamics simulation

**Fig. 7:**
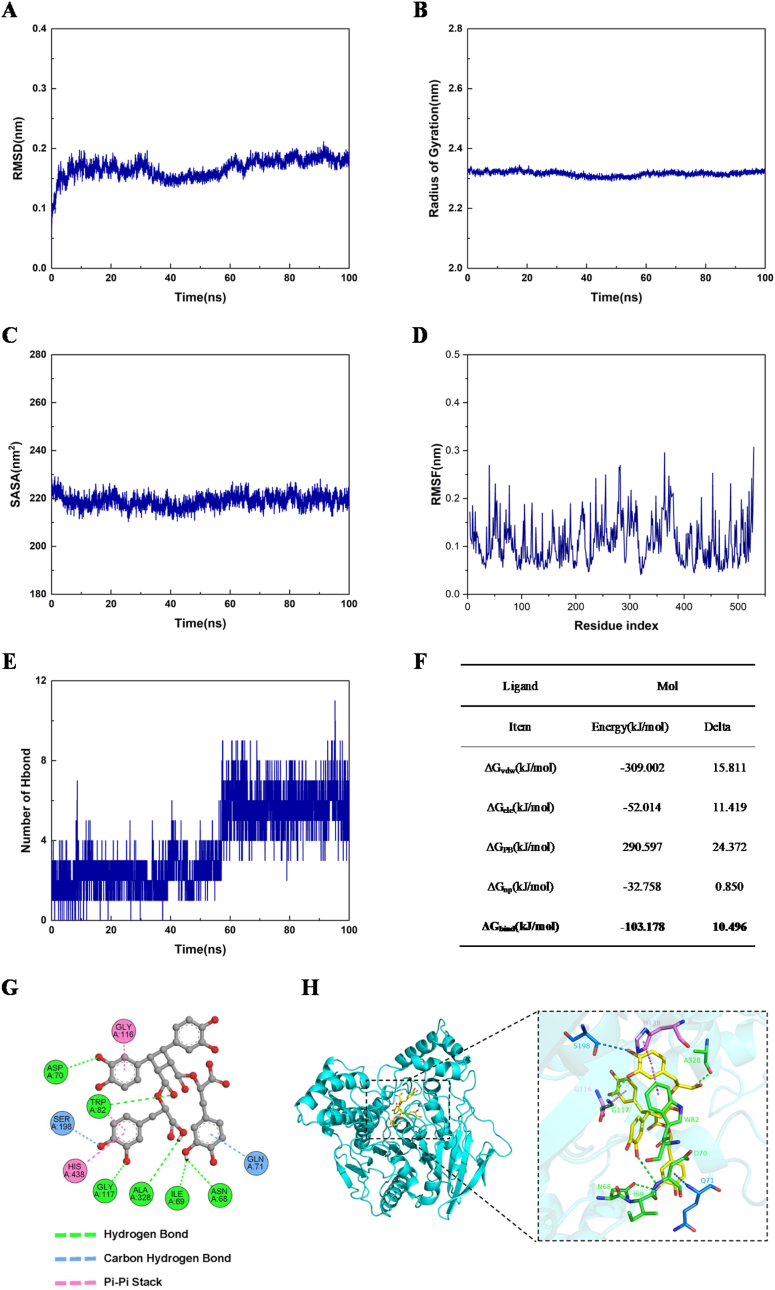
The molecular dynamics simulations of BChE-Sagerinic Acid. **A)** The variation of RMSD values with simulation time. **B)** The variation of turning radius with simulation time. **C)** The change of protein solvent accessible surface area (SASA) over simulation time. **D)** The RMSF values of all amino acid residues in the protein. **E)** The variation of hydrogen bond number with simulation time. **F)** The energy terms of the binding free energy between BChE and sagerinic acid. **G)** The binding mode of BChE and Sagerinic Acid in the system after the simulation ended (2D). **H)** The binding mode of BChE and Sagerinic Acid in the system after the simulation ended (3D).

To further elucidate the stability of the interaction between small molecules and proteins, the BChE–sagerinic acid complex system was selected for molecular dynamics simulation. The root-mean-square deviation (RMSD) is a widely used metric for quantifying the overall deviation of atomic coordinates across different conformational states relative to a reference structure, serving as a key indicator of system stability. As illustrated in Fig. 7A, the RMSD profile of all Cα atoms in the protein system was monitored throughout the simulation. The results show that the RMSD of the complex protein system remains consistently low, averaging 0.167 ± 0.016 nm. The value stabilizes rapidly during the simulation's initial phase. Subsequently, it maintains a stable trajectory with minimal fluctuations, indicating high structural integrity and system reliability over time. These observations suggest that the protein complex adopts a stable conformation under the simulated conditions.

The radius of gyration (Rg) serves as a key parameter for assessing the overall compactness of a protein structure. A consistent Rg value typically characterizes a stable protein fold, whereas significant changes in Rg reflect substantial structural rearrangements. As illustrated in Fig. 7B, the Rg of the protein–small molecule complex system exhibits only minor fluctuations throughout the simulation. The average Rg over the entire simulation period is 2.316 ± 0.008 nm, with minimal deviation. These small variations suggest that the conformation of the protein–ligand complex remains highly stable under the simulated conditions.

The solvent-accessible surface area (SASA) is a critical parameter for characterizing the dynamic behavior of protein structures and the stability of macromolecular interfaces. In this study, the structural stability of the protein complex was assessed by monitoring SASA fluctuations throughout MD simulations (Fig. 7C). The results show that the SASA value remained relatively stable, oscillating slightly around 219.037 ± 2.726 nm^2^, with a consistent kinetic trajectory. This minimal variation in SASA indicates that the overall conformation of the complex, as well as the subunit-binding interface, reached equilibrium, with no significant dissociation or structural reorganization observed. Therefore, it can be concluded that the intermolecular interactions stabilizing the complex were preserved during the simulation, underscoring the structural robustness of the multi-protein assembly.

The root-mean-square fluctuation (RMSF) values of each amino acid residue in the protein within the complex system reflect local changes in protein flexibility during the molecular dynamics simulation. As shown in Fig. 7D, the RMSF profile of the protein system indicates that, overall, the main structural regions exhibit relatively low RMSF values (typically below 0.3 nm), suggesting minimal atomic displacement and a high degree of structural stability. This implies that the protein's core domain maintains conformational integrity throughout the simulation, which is essential for preserving its proper three-dimensional fold and biological activity. In contrast, elevated RMSF values are observed in specific terminal segments and loop regions, indicating higher flexibility in these areas. Such dynamic characteristics may be functionally relevant, potentially facilitating protein–ligand interactions or conformational adjustments during regulatory processes. Collectively, the RMSF analysis demonstrates that the system has attained a stable conformational state during the equilibrium phase, with no significant perturbations detected in the protein backbone structure.

Hydrogen bonds play a critical role in the recognition and binding processes between proteins and small molecules, representing one of the key non-covalent interactions that contribute to the structural stability and specificity of macromolecular complexes. Stable, persistent hydrogen bonds not only enhance the binding affinity between ligands and receptors but also influence protein conformational changes and dynamic equilibria. As illustrated in Fig. 7E, this study analyzed the temporal evolution of hydrogen bond counts during molecular dynamics simulations of the protein–ligand complex. The results demonstrate that hydrogen bonds between the protein and the small molecule remained consistently stable throughout the simulation, with an average count of 3.754, showing a slight increasing trend over time. This pattern indicates a progressive stabilization of the protein–ligand interaction, suggesting increasingly tighter binding. The sustained hydrogen bond network plays a crucial role in maintaining the overall conformational integrity of the complex, thereby supporting its biological function.

In this study, the MM/PBSA method was employed to calculate the binding free energy of the protein–ligand complex over the 80–100 ns time interval, during which the system's RMSD remained relatively stable. The results are summarized in Fig. 7F. The calculated total binding free energy between the small molecule and the protein was −103.178 kJ/mol, indicating a strong binding propensity. Energy decomposition analysis revealed that the sum of electrostatic interaction energies—comprising vacuum electrostatic interactions (ΔGele) and polar solvation energy (ΔGPB)—was +238.583 kJ/mol, whereas the non-polar interaction energy—including van der Waals interactions (ΔGvdw) and non-polar solvation free energy (ΔGnp)—was −341.760 kJ/mol. These findings demonstrate that non-polar interactions dominate the overall binding free energy and make the primary contribution to the binding process. This suggests that hydrophobic interactions are the principal driving force underlying the protein–ligand association, with electrostatic interactions serving as a secondary, modulatory influence. Collectively, the hydrophobic effect plays a critical role in stabilizing the complex, thereby providing a solid theoretical foundation for elucidating the molecular binding mechanism.

Following the simulation, we systematically analyzed the binding mode between the protein and the small molecule within the system. The results are presented in Fig. 7. As illustrated in the 2D interaction diagram (Fig. 7G), the small molecule forms six conventional hydrogen bonds with the amino acid residues Asp70, Trp82, Gly117, Ala328, Ile69, and Asn68 of the protein. Additionally, it engages in weak CH···X hydrogen bonding interactions with Ser198 and Gln71 located in the vicinity of the binding pocket. Furthermore, π-π stacking interactions are observed with His438, Gly116, and Trp82. The 3D structural analysis (Fig. 7H) confirms that the small molecule is stably accommodated within the protein binding site. These findings indicate that the protein maintains a stable complex with the small molecule through multiple non-covalent interactions.

Despite the promising insights gained from our molecular docking and dynamics simulations, it is important to acknowledge the inherent limitations of these computational approaches. The predictions of binding affinity and complex stability are based on theoretical models and simplified force fields, which may not fully capture the complexity of biological systems. Furthermore, the absence of experimental validation for the predicted protein–ligand interactions and binding modes necessitates caution in interpreting these results. Future studies involving site-directed mutagenesis, biophysical binding assays, or structural biology techniques are warranted to confirm the proposed mechanisms of action.

## 4 Conclusion

To our knowledge, this is the initial report detailing the phytochemical profile and biological effects of *P. hedgei*. The findings indicated an abundance of phenolics, with rosmarinic acid predominantly present in both the aerial parts and roots extracts. The root extracts demonstrated superior antioxidant properties. Additionally, the ethyl acetate extracts showed the most potent cytotoxic effects on HELA and HCT-116 cell lines. These results suggest that *P. hedgeii* might serve as a valuable source of active ingredients for various cosmetic and pharmaceutical uses. While the network pharmacology and molecular docking results are predictive, they offer a robust hypothesis and a prioritized roadmap for future research. Further studies are warranted to experimentally validate the proposed targets and pathways, including the isolation of bioactive compounds, *in vivo* validation, and transcriptomic/proteomic profiling.

## Consent to participate

Not applicable.

## Ethical approval

Not applicable.

## Author contributions

Conceptualization, ES, SY, BHA, EJLM, GZ; methodology, ES, SY, BHA, EJLM, IK, MC, IY, IG, GZ.; software, YW, ML, GZ; validation, EY, ML, GZ; formal analysis, GZ; investigation, ES, SY, BHA, IK, MC, YW, ML, GZ; resources, EY, GZ; data curation, GZ writing—original draft preparation, ES, SY, BHA, YW, ML, GZ; writing—review and editing, EJLM, YW, ML, GZ.; visualization, YW, ML, GZ.; supervision, ML, GZ.; project administration, GZ.; funding acquisition, GZ. All authors have read and agreed to the published version of the manuscript.

## Code availability

Not applicable.

## Consent for publication

Not applicable.

## Funding

This research received no external funding.

## Declaration of competing interest

The authors declare the following financial interests/personal relationships which may be considered as potential competing interests:

The authors declare that there are no conflicts of interest.

## Data Availability

Data will be made available on request.
