# Supplementary material for: Unleashing the biological power and chemical profile of Paracaryum hedgei extracts based on chromatographic, in vitro, and bioinformatic tools
Source: Biochem Biophys Rep. 2025 Dec 14;45:102408. doi: 10.1016/j.bbrep.2025.102408 (PMC12765141; doi:10.1016/j.bbrep.2025.102408)
Supplement: Multimedia component 1 [file mmc1.docx]

**Phytochemical analysis by HPLC-ESI-Q-TOF-MS**

The characterization of the compounds was carried out by high-performance liquid chromatography with electrospray ionization and a quadrupole-time-of-flight mass spectrometer (HPLC-ESI-Q-TOF-MS). For the analyses, 5 mg of each dried extract was dissolved in 1 mL of methanol (except the aqueous extract, which was re-dissolved in 1 mL of 10% MeOH) and filtered through 0.45 µm membrane filters. An Agilent 1200 (Agilent Technologies, Santa Clara, CA, USA) equipped with an Agilent 6530B Q-TOF MS was used; a Luna Omega Polar C_18_ column of 150 x 3.0 mm (5 µm particle size) with a Polar C18 Security Guard cartridge of 4 × 3.0 mm (Phenomenex, Torrance, CA, USA) was utilized for the HPLC separation.

The HPLC gradient elution program (at a flow rate of 0.4 mL min^-1^) was developed using mobile phases of water + formic acid 0.1 % v/v (eluent A) and acetonitrile (eluent B). The elution program was: 10-25% B in 0-25 min, 25% B in 25-30 min, 25-50% B in 30-40 min, 50-100% B in 40-42 min; then, eluent B was returned to 10% with a 7-min stabilization time. To obtain the MS and MS/MS spectra, the mass spectrometer was operated in the negative ion mode using an orthogonal ESI source (Agilent Dual ESI, Santa Clara, CA, USA). An auto-tuning is performed before analysis with an auto-tuning solution (Esi-L Low concentration tuning mix) from Agilent Technologies. Reference masses were used at m/z 112.9855 (purine) and 1033.98 (hexakis). The parameters used were: capillary voltage, 3500 V; nebulizer pressure of 45 psi; drying gas flow rate, 10 L/min; gas temperature, 325 °C; skimmer voltage, 60 V; fragmentor voltage, 140 V. The MS and Auto MS/MS modes were set to acquire m/z values ranging between 50-1200, at a scan rate of 2 and 3 spectra per second, respectively. The MS mode was used for qualitative and quantitative purposes, whereas the auto MS/MS was used for the characterization (MS fragmentation pattern). Agilent Mass Hunter Qualitative analysis software version B.06.00 was used for post-acquisition data processing. Molecular formulas, errors (ppm), and fragment ions were obtained by the tool Generate Formulas using the Auto MS/MS algorithm. For the quantitative discussion, Extracted Ion Chromatograms (EICs) in MS mode were obtained for each compound at its deprotonated molecular ion (using a symmetric expansion of ±5 ppm), and integrated peak areas were used to compare phytochemical profiles between leaves, as well as the extraction efficiency of the different solvents. Relative standard deviations were lower than 5% in all cases.

For identification purposes, analytical standards were injected individually as external standards, and their retention times, accurate mass (in MS mode), and fragmentation patterns (in MS/MS mode at collision energies of 10, 20, and 40 V) were used to identify the same compounds in the extracts. Regarding derivatives, such as flavonoid glycosides, the MS/MS fragmentation of the aglycones was used to confirm their identity, whereas the observed neutral losses were used to characterize the attached moieties (such as sugars). The METLIN database was also used to confirm the identification.

*Assays for Total Phenolic and Flavonoid Contents*

The total phenolic content was determined by employing the methods given in the literature with some modiﬁcation. Sample solution (0.25 mL) was mixed with diluted Folin–Ciocalteu reagent (1 mL, 1:9, v/v) and shaken vigorously. After 3 min, Na_2_CO_3_ solution (0.75 mL, 1%) was added and the sample absorbance was read at 760 nm after a 2 h incubation at room temperature. The total phenolic content was expressed as milligrams of gallic acid equivalents (mg GAE/g extract)[1].

The total ﬂavonoid content was determined using the AlCl_3_ method. Brieﬂy, sample solution (1 mL) was mixed with the same volume of aluminum trichloride (2%) in methanol. Similarly, a blank was prepared by adding sample solution (1 mL) to methanol (1 mL) without AlCl_3_. The sample and blank absorbances were read at 415 nm after a 10 min incubation at room temperature. The absorbance of the blank was subtracted from that of the sample. Rutin was used as a reference standard and the total ﬂavonoid content was expressed as milligrams of rutin equivalents (mg RE/g extract) [1].

*Determination of Antioxidant and Enzyme Inhibitory Effects*

Antioxidant (DPPH and ABTS radical scavenging, reducing power (CUPRAC and FRAP), phosphomolybdenum and metal chelating (ferrozine method)) and enzyme inhibitory activities (cholinesterase (Elmann’s method), tyrosinase (dopachrome method), α-amylase (iodine/potassium iodide method), α -glucosidase (chromogenic PNPG method) and pancreatic lipase (*p*-nitrophenyl butyrate (p-NPB) method) were determined using the methods previously described by Uysal et al. [1] and Grochowski et al. [2]

For the DPPH (1,1-diphenyl-2-picrylhydrazyl) radical scavenging assay: Sample solution was added to 4 mL of a 0.004% methanol solution of DPPH. The sample absorbance was read at 517 nm after a 30 min incubation at room temperature in the dark. DPPH radical scavenging activity was expressed as milligrams of trolox equivalents (mg TE/g extract).

For ABTS (2,2′-azino-bis(3-ethylbenzothiazoline) 6-sulfonic acid) radical scavenging assay: Brieﬂy, ABTS+ was produced directly by reacting 7 mM ABTS solution with 2.45 mM potassium persulfate and allowing the mixture to stand for 12–16 in the dark at room temperature. Prior to beginning the assay, ABTS solution was diluted with methanol to an absorbance of 0.700 ± 0.02 at 734 nm. Sample solution was added to ABTS solution (2 mL) and mixed. The sample absorbance was read at 734 nm after a 30 min incubation at room temperature. The ABTS radical scavenging activity was expressed as milligrams of trolox equivalents (mg TE/g extract).

For CUPRAC (cupric ion reducing activity) activity assay: Sample solution was added to premixed reaction mixture containing CuCl_2_ (1 mL, 10 mM), neocuproine (1 mL, 7.5 mM) and NH_4_Ac buffer (1 mL, 1 M, pH 7.0). Similarly, a blank was prepared by adding sample solution (0.5 mL) to premixed reaction mixture (3 mL) without CuCl_2_ . Then, the sample and blank absorbances were read at 450 nm after a 30 min incubation at room temperature. The absorbance of the blank was subtracted from that of the sample. CUPRAC activity was expressed as milligrams of trolox equivalents (mg TE/g extract).

For FRAP (ferric reducing antioxidant power) activity assay: Sample solution was added to premixed FRAP reagent (2 mL) containing acetate buffer (0.3 M, pH 3.6), 2,4,6-tris(2-pyridyl)-S-triazine (TPTZ) (10 mM) in 40 mM HCl and ferric chloride (20 mM) in a ratio of 10:1:1 (v/v/v). Then, the sample absorbance was read at 593 nm after a 30 min incubation at room temperature. FRAP activity was expressed as milligrams of trolox equivalents (mg TE/g extract).

For phosphomolybdenum method: Sample solution was combined with 3 mL of reagent solution (0.6 M sulfuric acid, 28 mM sodium phosphate and 4 mM ammonium molybdate). The sample absorbance was read at 695 nm after a 90 min incubation at 95 °C. The total antioxidant capacity was expressed as millimoles of trolox equivalents (mmol TE/g extract).

For metal chelating activity assay: Brieﬂy, sample solution was added to FeCl_2_ solution (0.05 mL, 2 mM). The reaction was initiated by the addition of 5 mM ferrozine (0.2 mL). Similarly, a blank was prepared by adding sample solution (2 mL) to FeCl_2_ solution (0.05 mL, 2 mM) and water (0.2 mL) without ferrozine. Then, the sample and blank absorbances were read at 562 nm after 10 min incubation at room temperature. The absorbance of the blank was sub-tracted from that of the sample. The metal chelating activity was expressed as milligrams of EDTA (disodium edetate) equivalents (mg EDTAE/g extract).

For Cholinesterase (ChE) inhibitory activity assay: Sample solution (was mixed with DTNB (5,5-dithio-bis(2-nitrobenzoic) acid, Sigma, St. Louis, MO, USA) (125 µL) and AChE (acetylcholines-terase (Electric ell acetylcholinesterase, Type-VI-S, EC 3.1.1.7,Sigma)), or BChE (butyrylcholinesterase (horse serum butyrylcholinesterase, EC 3.1.1.8, Sigma)) solution (25 μL) in Tris–HCl buffer (pH 8.0) in a 96-well microplate and incubated for 15 min at 25 °C. The reaction was then initiated with the addition of acetylthiocholine iodide (ATCI, Sigma) or butyrylthiocholine chloride (BTCl, Sigma) (25 μL). Similarly, a blank was prepared by adding sample solution to all reaction reagents without enzyme (AChE or BChE) solution. The sample and blank absorbances were read at 405 nm after 10 min incubation at 25 °C. The absorbance of the blank was subtracted from that of the sample and the cholinesterase inhibitory activity was expressed as galanthamine equivalents (mgGALAE/g extract).

For Tyrosinase inhibitory activity assay: Sample solution was mixed with tyrosinase solution (40 μL, Sigma) and phosphate buffer (100 μL, pH 6.8) in a 96-well microplate and incubated for 15 min at 25 °C. The reaction was then initiated with the addition of L-DOPA (40 μL, Sigma). Similarly, a blank was prepared by adding sample solution to all reaction reagents without enzyme (tyrosinase) solution. The sample and blank absorbances were read at 492 nm after a 10 min incubation at 25 °C. The absorbance of the blank was subtracted from that of the sample and the tyrosinase inhibitory activity was expressed as kojic acid equivalents (mgKAE/g extract).

For α-amylase inhibitory activity assay: Sample solution was mixed with α-amylase solution (ex-porcine pancreas, EC 3.2.1.1, Sigma) (50 μL) in phosphate buffer (pH 6.9 with 6 mM sodium chloride) in a 96-well microplate and incubated for 10 min at 37 °C. After pre-incubation, the reaction was initiated with the addition of starch solution (50 μL, 0.05%). Similarly, a blank was prepared by adding sample solution to all reaction reagents without enzyme (α-amylase) solution. The reaction mixture was incubated 10 min at 37 °C. The reaction was then stopped with the addition of HCl (25 μL, 1 M). This was followed by addition of the iodine-potassium iodide solution (100 μL). The sample and blank absorbances were read at 630 nm. The absorbance of the blank was subtracted from that of the sample and the α-amylase inhibitory activity was expressed as acarbose equivalents (mmol ACE/g extract).

For α-glucosidase inhibitory activity assay: Sample solution was mixed with glutathione (50 µL), α-glucosidase solution (from Saccharomyces cerevisiae, EC 3.2.1.20, Sigma) (50 µL) in phosphate buffer (pH 6.8) and PNPG (4-N-trophenyl-α-D-glucopyranoside, Sigma) (50 µL) in a 96-well microplate and incubated for 15 min at 37 °C. Similarly, a blank was prepared by adding sample solution to all reaction reagents without enzyme (α-glucosidase) solution. The reaction was then stopped with the addition of sodium carbonate (50 µL, 0.2 M). The sample and blank absorbances were read at 400 nm. The absorbance of the blank was subtracted from that of the sample and the α-glucosidase inhibitory activity was expressed as acarbose equivalents (mmol ACE/g extract).

**References**

1. Uysal, S., et al., *Cytotoxic and enzyme inhibitory potential of two Potentilla species (P. speciosa L. and P. reptans Willd.) and their chemical composition.* Frontiers in pharmacology, 2017. **8**: p. 290.

2. Grochowski, D.M., et al., *In vitro enzyme inhibitory properties, antioxidant activities, and phytochemical profile of Potentilla thuringiaca.* Phytochemistry Letters, 2017. **20**: p. 365-372.
